# Apical integrins as a switchable target to regulate the epithelial barrier

**DOI:** 10.1242/jcs.263580

**Published:** 2024-12-20

**Authors:** Raven J. Peterson, Ryan C. Reed, Colin R. Zamecnik, Marwa A. Sallam, Joel A. Finbloom, Francisco J. Martinez, Joshua M. Levy, Aekkacha Moonwiriyakit, Tejal A. Desai, Michael Koval

**Affiliations:** ^1^ Division of Pulmonary, Allergy, Critical Care and Sleep Medicine, Department of Medicine, Emory University School of Medicine, Atlanta, GA 30322, USA; ^2^ Department of Bioengineering and Therapeutic Sciences, University of California San Francisco, San Francisco, CA 94158, USA; ^3^ School of Engineering, Brown University, Providence, RI 02912, USA; ^4^ Department of Otolaryngology, Emory University School of Medicine, Atlanta, GA 30322, USA; ^5^ National Institute on Deafness and Other Communication Disorders, National Institutes of Health, Bethesda, MD 20892, USA; ^6^ Chakri Naruebodindra Medical Institute, Faculty of Medicine Ramathibodi Hospital, Mahidol University, Bang Phli, Samut Prakan, 10540, Thailand; ^7^ Department of Cell Biology, Emory University School of Medicine, Atlanta, GA 30322, USA

**Keywords:** Tight junction, Integrin, Claudin, ZO-1, Epithelium, Paracellular permeability

## Abstract

Tight junctions regulate epithelial barrier function and have been shown to be influenced by multiple classes of proteins. Apical integrins have been identified as potential regulators of epithelial barrier function; however, only indirect approaches have been used to measure integrin regulation of the epithelial barrier. Here, we used polymeric nanowires conjugated with anti-integrin β1 antibodies to specifically target apically localized integrins in either their closed or open conformation. Barrier regulation by apical integrins was found to be conformation specific. Nanowires targeting integrins in the closed conformation increased epithelial permeability and caused zonula occludens-1 (ZO-1, also known as TJP1) to change from a linear to a ruffled morphology. Claudin-2 and claudin-4 colocalized with ZO-1 and were also ruffled; however, claudin-1 and claudin-7 remained linear. Ruffling was dependent on myosin light chain kinases (MLCKs) and Rho kinases (ROCKs). Conversely, targeting integrins in the open conformation decreased epithelial permeability and made junctions more linearized. Anti-integrin β1 nanowires differentially affected actin and talin (analyzed using pan-talin antibodies), depending on whether they contained activating or inhibitory antibodies. Thus, apical integrins can act as a conformation-sensitive switch that regulates epithelial barrier function.

## Teaser

**Summary:** Nanowires specifically targeting apical active or inactive integrins were used to determine that integrins differentially regulate tight junction morphology and epithelial barrier function depending on activation state.

## INTRODUCTION

A key function of epithelial cells is creating a physiologically active barrier that partitions distinct tissue environments. Epithelial cells are polarized on an apical–basolateral axis established by intercellular contacts mediated by the apical junctional complex (AJC) and cell attachment to the extracellular matrix. The AJC creates an adhesive meshwork between cells that includes polarity complex proteins, adherens junctions and tight junctions (Buckley and Turner, 2018; Rouaud et al., 2020). The main role of tight junctions is to regulate paracellular permeability, primarily by claudin family transmembrane proteins, which form paracellular ion channels. A stable tight junction also includes cytosolic scaffold zonula occludens (ZO) proteins, which crosslink integral tight junction proteins with the actin cytoskeleton and polarity complex proteins (Quiros and Nusrat, 2014; Sun et al., 2022). The expression and organization of tight junction proteins is tissue specific, allowing for organ-specific permeability (Lynn et al., 2020).

Epithelial cell polarity is also defined by integrins, which are α–β heterodimeric transmembrane proteins that bind to the extracellular matrix to regulate the actin cytoskeleton, cell signaling and mechanosensing (Humphries et al., 2019; Hynes, 2002; Kadry and Calderwood, 2020; Schwartz, 2010; Sun et al., 2019). Recently, it has become appreciated that apically oriented integrins can also regulate cell function (Peterson and Koval, 2021). In addition to their classical role as receptors for extracellular matrix proteins, there have been several examples of integrins interacting with adherens junction proteins (Wang et al., 1999) and tight junction proteins (Fredriksson et al., 2015; Lu et al., 2015), suggesting that integrins might play a role in regulating barrier function in epithelial cells (Starchenko et al., 2017). In fact, the proximity of apically localized integrins to tight junction proteins allows for barrier disruption by bacterial infection (Tafazoli et al., 2000).

There are emerging data that suggest a possible role for apical integrins in regulating epithelial barrier function. For instance, contact between apical integrins and large overlays such as collagen hydrogels (Garbi et al., 1996), nanostructured thin films (Huang et al., 2020; Kam et al., 2013; Stewart et al., 2017; Walsh et al., 2015) and anionic nanoparticles (Lamson et al., 2020) has been correlated with increased barrier leak as measured by increased permeability of ions and large molecules, along with corresponding changes in the morphology and localization of tight junction proteins. Although these data raise the possibility that integrin clustering and stimulation can regulate epithelial barrier function, previous research is limited by the fact that matrix overlays and nanostructured films contact the entire apical monolayer rather than specifically probing integrins.

To overcome this hurdle, we used a discrete nanowire platform (Finbloom et al., 2020; Zamecnik et al., 2020, 2017) decorated with antibodies (anti-integrin nanowires) as a multivalent platform with the capacity to specifically bind and stimulate apically oriented integrins. There are many well characterized anti-integrin antibodies that can be conjugated to form anti-integrin nanowires, including classes of monoclonal antibodies (mAbs) that are sensitive to integrin conformation and activity state (Byron et al., 2009; Spiess et al., 2018; Su et al., 2016; Takada and Puzon, 1993). This is critically important, since integrins can assume several functionally distinct conformations, ranging from an inactive bent state to an active extended state that enables high-affinity ligand binding (Li and Springer, 2017; Luo et al., 2007; Su et al., 2016; Takagi et al., 2002; Ye et al., 2012).

There are two primary classes of integrin-engaging mAbs, distinguished by the integrin confirmation to which they bind. The first class of antibodies are the ligand-induced binding site (LIBS) mAbs, which bind epitopes that are only exposed when integrins are in the open conformation (Byron et al., 2009; Mould et al., 1995; Su et al., 2016). These antibodies can be used to detect populations of integrins in the open conformation (Bazzoni et al., 1995; Tsuchida et al., 1997), and they promote increased ligand binding by stabilizing the integrin in the active state (Luque et al., 1996). On the other hand, blocking antibodies, often called ligand-attenuated binding site (LABS) mAbs, prevent ligand binding by allosteric regulation of the ligand binding site (Byron et al., 2009; Mould et al., 1996; Su et al., 2016; Takagi and Springer, 2002). Using nanowires conjugated with either a LIBS mAb or LABS mAb allows us to measure the impact of a multivalent ligand targeting apical integrins with different activity states on the regulation of epithelial barriers. Multivalent ligands are particularly relevant to elucidate roles for integrins in cell function, since they generally function as multiprotein complexes (e.g. focal adhesions; Horton et al., 2016). Recent evidence also supports the segregation of inactive and active integrins into distinct subdomains, which can influence their function (Spiess et al., 2018). Nanowires of different sizes and aspect ratios provide multivalent ligands that can specifically manipulate integrin organization.

We show in this study that targeting apical integrins with nanowires conjugated to LIBS or LABS anti-integrin antibodies causes differential changes in the epithelial barrier as measured by changes in the morphology and permeability of tight junctions and adherens junctions. This suggests that stimulation of apical integrins is sufficient to regulate components of the AJC and that the conformation state of clustered apical integrins plays a key role in how integrins regulate the epithelial barrier. The functional implications of the ability to control epithelial barrier function using integrin targeted nanowires are also discussed, including increasing permeability to promote drug delivery or decreasing permeability to promote tissue integrity.

## RESULTS

### Multimeric engagement of apical integrin β1 by blocking antibodies induces tight junction ruffling

**Fig. 1.**
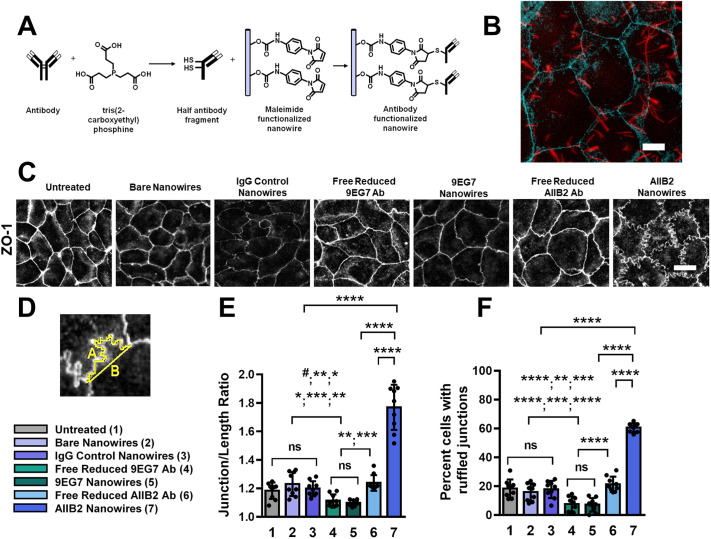
**Targeting apical integrins with AIIB2 nanowires produces a ruffled ZO-1 morphology.** (A) Schematic of the general reduction and conjugation reactions that generate the antibody-decorated nanowires used in these experiments. (B) Representative immunofluorescence image of bare nanowires (labeled with Nile Red; red) on Caco-2 cells labeled with anti-ZO-1 antibodies (cyan). Scale bar: 10 μm. Image representative of five experiments. (C) Representative immunofluorescence images of Caco-2 cells labeled with anti-ZO-1 antibodies either untreated or 2 h after treatment with bare nanowires, IgG control nanowires, free reduced antibody (9EG7 or AIIB2), or anti-integrin nanowires (9EG7 or AIIB2). Scale bar: 30 μm. (D) Changes in ZO-1 morphology are quantified by measuring the ratio of the traced actual length between tricellular junctions (trace A) and the linear distance between those same junctions (trace B). (E,F) Quantification of junction length ratios (E) and percent of cells in a field of view with one or more ruffled junction (F) for each treatment displayed as mean±s.d. (*n*=3 fields of view from three slides per condition; 25 measurements per field of view). Treatment key on left (Ab, antibody). Significance determined by one-way ANOVA with Fisher's LSD test. (E) ^#^*P*=0.66 (1 versus 4); **P=0.003 (2 versus 4); *P=0.028 (3 versus 4); *P=0.025 (1 versus 5); ***P=0.0008 (2 versus 5); **P=0.009 (3 versus 5); **P=0.0015 (4 versus 6); ***P=0.0004 (5 versus 6); ****P<0.0001 (7 versus all); ns, not significant. (F) ****P<0.0001 (1 versus 4); **P=0.016 (2 versus 4); ***P=0.002 (3 versus 4); ****P<0.0001 (1 versus 5); ***P=0.0008 (2 versus 5); ****P<0.0001 (3 versus 5); ****P<0.0001 (4 versus 6, 5 versus 6, 7 versus all); ns, not significant.

We have previously observed that when the apical surface of epithelial cells is placed in contact with a film engineered with specific nanotopography, this causes ZO-1 (also known as TJP1) to assume a ruffled morphology accompanied by increased paracellular leak (Huang et al., 2020; Kam et al., 2013; Stewart et al., 2017; Walsh et al., 2015). Experiments using anti-integrin blockade prior to application of nanostructured films inhibits tight junction ruffling, implicating a role for integrins (Walsh et al., 2015). However, nanostructured films engage the entire apical plasma membrane surface. To directly determine whether apical integrins influence tight junctions, we produced a discrete nanowire platform that can be derivatized with antibodies to specifically target pools of apically localized integrin β1 (Fig. 1A). Because integrin conformation state is crucial for integrin activity, we produced anti-integrin nanowires that target integrin β1 in either the closed state or extended state with well characterized mAbs. To do this, we used blocking mAb AIIB2, a LABS antibody that binds integrin β1 by inhibiting ligand binding in both the extended and bent conformation (Hall et al., 1990; Park et al., 2008; Spiess et al., 2018; Werb et al., 1989), and an activating mAb, 9EG7, which is a LIBS antibody that binds the EGF repeats when integrin β1 is in the extended conformation and promotes ligand binding (Bazzoni et al., 1995; Byron et al., 2009). The antibodies were selectively reduced using tris(2-carboxyethyl)phosphine to produce half-antibody fragments containing free sulfhydryl groups. These half antibodies had the capacity to be covalently conjugated to polycaprolactone (PCL) nanowires that had maleimide groups exposed on the particle surfaces (Fig. 1A).

Cells were treated with anti-integrin nanowires (Fig. 1B) or control preparations for 2 h, and then quantitative ZO-1 immunostaining was used to determine the effect of targeting apically localized integrins on tight junction morphology (Fig. 1C). The most striking result was that cells treated with AIIB2 nanowires acquired a robust and quantifiable ruffled ZO-1 morphology, as characterized by a junction length ratio significantly greater than 1 (1.7±0.2, mean±s.d.; *n*=9) and a high percentage of cells showing ruffled junctions (60.7±3.0%, mean±s.d.; *n*=9) (Fig. 1D–F). By contrast, soluble reduced AIIB2 had little effect on tight junction morphology. Moreover, treating cells with either soluble reduced 9EG7 antibody or 9EG7 nanowires had the reverse effect on ZO-1: tight junctions became more linear and there was a significantly smaller proportion of cells exhibiting a ruffled morphology (7.7±5.2% for 9EG7 antibody, 7.1±4.7% for 9EG7 nanowires; *n*=9) compared to untreated controls (18.4±6.3%; *n*=9) (Fig. 1E,F). None of these treatments had an impact on cell viability (Fig. S1A). Junction ruffling induced by AIIB2 nanowires did not alter the apical–lateral localization of junctions, as demonstrated by localization of ZO-1 in the *xz* and *yz* plane using three-dimensional confocal immunofluorescence microscopy (Fig. S1B). Also, the ability of AIIB2 to cause ZO-1 ruffling depended on the aspect ratio of the antibody-conjugated particle, since microspheres conjugated to AIIB2 had little effect on ZO-1 morphology (Fig. S2).

To visualize where cell-associated nanowires were located in relation to ruffled tight junctions, we imaged cells challenged with fluorescently labeled AIIB2 nanowires (Fig. S3). A significantly greater proportion of the cell-associated AIIB2 nanowires were junction associated, as compared to the localization of cell-associated bare nanowires. Of the AIIB2 nanowires that were localized to cell–cell contact sites, significantly more AIIB2 nanowires were adjacent to ruffled tight junctions as opposed to being in direct contact with ruffled tight junctions. The correlation of AIIB2 nanowire localization relative to ruffled junctions suggests that the integrin regulation of tight junctions was due to action at a distance as opposed to local action mediated by direct contact with the junctions themselves to cause ruffling.

**Fig. 2.**
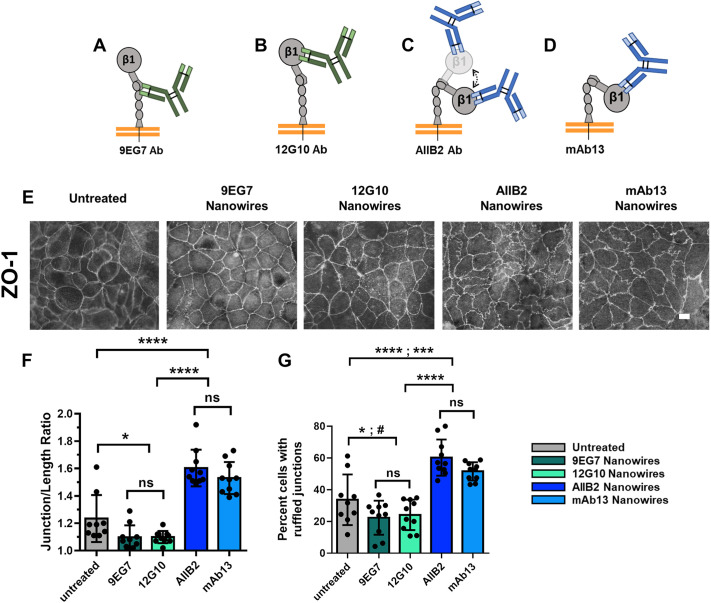
**Targeting apical integrins with blocking nanowires produces a ruffled ZO-1 morphology.** (A) The 9EG7 activating mAb (green) binds to the EGF domain locking an extended conformation. (B) The 12G10 activating mAb (green) binds to the βA domain locking an extended conformation. (C) The AIIB2 blocking mAb (blue) binds to the ligand binding site of the βA domain, irrespective of conformation. (D) The mAb13 blocking mAb (blue) binds to the βA domain locking a bent configuration. Ab, antibody; β1, integrin β1. (E) Representative immunofluorescence images of Caco-2 cells labeled with anti-ZO-1 antibodies either untreated or 2 h after treatment with the indicated anti-integrin nanowires. Scale bar: 10 μm. (F,G) Quantification of junction length ratios (F) and percent of cells in a field of view with one or more ruffled junctions (G) for each treatment displayed as mean±s.d. (*n*=4 or 5 fields of view from two slides per condition; 15 measurements per field). Treatment key on right. Significance determined by one-way ANOVA with Fisher's LSD test. (F) *P=0.015 (untreated versus 9EG7); *P=0.016 (untreated versus 12G10); ****P<0.0001; ns, not significant. (G) *P=0.031 (untreated versus 9EG7); ^#^*P*=0.066 (untreated versus 12G10); ***P=0.001 (untreated versus mAb13); ****P<0.0001; ns, not significant.

To determine whether our findings translated to other well characterized anti-integrin β1 antibodies, we produced nanowires conjugated to either mAb13, an inhibitory LABS antibody that specifically locks the headpiece in a closed, bent configuration, or 12G10, an activating LIBS antibody that recognizes an open headpiece (Mould et al., 1996; Su et al., 2016). As shown in Fig. 2, mAb13 nanowires induced tight junction ruffling to an extent that was comparable to AIIB2 nanowires. Conversely, 12G10 nanowires decreased tight junction ruffling to an extent that was comparable to 9EG7 nanowires. Taken together, these data demonstrate that anti-integrin antibodies have the capacity to alter tight junction morphology and that the nature of the alteration depends on whether the antibody has an inhibitory or activating effect, the substrate valency, and the substrate aspect ratio.

### Time course and reversibility of anti-integrin nanowire–cell interactions

**Fig. 3.**
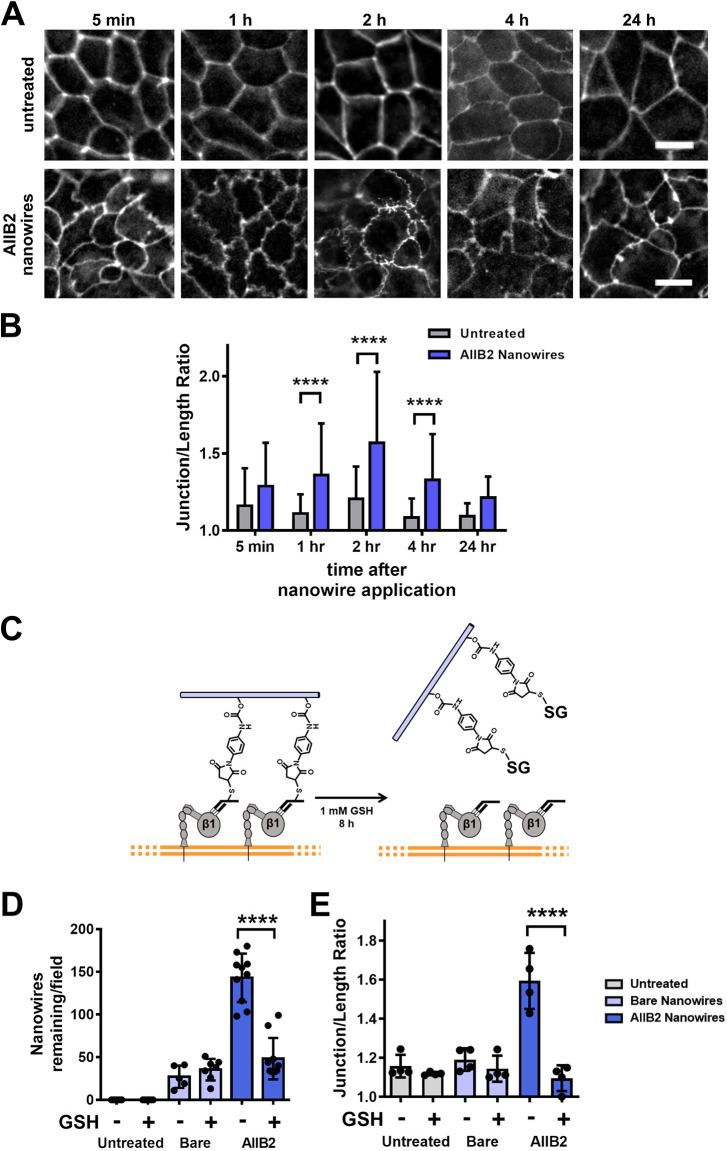
**Time course and reversibility of the effects of AIIB2 nanowires on tight junctions.** (A) Representative immunofluorescence images of Caco-2 cells labeled with anti-ZO-1 antibodies and fixed at multiple time points after treatment with AIIB2 nanowires. Scale bars: 10 μm. (B) Quantitation of junction length ratios for both treatments at each time point showed that significant ZO-1 ruffling occurred 1 h after application of nanowires. Data displayed as mean junction length ratio+s.d. (*n*=50 measurements per treatment per time point). Significance was determined by two-way ANOVA with Bonferroni's correction for multiple comparisons. ****P<0.0001. (C) Schematic of the GSH competition assay where cells were incubated with AIIB2 nanowires for 2 h, followed by addition of 1 mM GSH, which cleaves antibody conjugates off the wires over an 8 h incubation period. β1, integrin β1. (D) Quantitation of the number of cell-associated nanowires in a field of view for each treatment. Each data point represents a count for a single field of view, with mean±s.d. indicated (*n*=5–7 fields of view for untreated and bare nanowire-treated samples; *n*=10 fields of view for AIIB2 nanowire-treated samples). (E) Quantification of junction length ratios for each treatment. Each data point represents the average of a single field of view, with mean±s.d. indicated (*n*=4 fields of view; 15 measurements per field). Significance determined by two-way repeated measures ANOVA with Bonferroni's correction for multiple comparisons. ****P<0.0001.

We next measured the effect of AIIB2 nanowires on junction morphology over time, using ZO-1 localization as a measure of tight junction ruffling. Contact with AIIB2 nanowires induced a significant increase in tight junction ruffling at 1 h after application, although some subtle changes in tight junction morphology were observed as early as 5 min after application (Fig. 3A,B). At 1 h after AIIB2 nanowire application, the junction length ratio for treated cells was 1.4±0.3 (mean±s.d.; *n*=50) as opposed to 1.1±0.1 (*n*=50) for untreated cells. The morphologic changes induced by AIIB2 nanowires lasted for 24 h, although the extent of ruffling began to decrease at 4 h after nanowire application.

Previously published work has determined that decreases in transepithelial electrical resistance (TER) and ruffled ZO-1 morphology induced by apical contact with nanostructured films are reversed 24 h after the removal of the films (Kam et al., 2013). A challenge with assessing the reversibility of AIIB2 nanowires is that the cell–anti-integrin nanowire interaction is fairly stable over an extended period (Fig. 3A,B), suggesting that removing anti-integrin nanowires from cells could not be accomplished simply by washing cell monolayers. Thus, to assess whether the anti-integrin nanowire-induced changes to the epithelial barrier are reversible, we used glutathione (GSH) to induce maleimide–thiol exchange and replace the half-antibody fragments covalently attached to the maleimide handle on the nanowires (Baldwin and Kiick, 2011; Shen et al., 2012). This results in GSH-bound nanowires and half-antibody fragments that remain bound to apical integrins (Fig. 3C).

For the GSH competition assay, we first treated cells with AIIB2 nanowires for 2 h followed by replacement with either control medium or medium with 1 mM GSH for an 8 h additional incubation. With this assay, we observed that the GSH-treated samples had significantly fewer cell-associated AIIB2 nanowires in each field of view as compared to AIIB2 nanowire-treated samples that were not further treated with GSH (Fig. 3D). Additionally, cells treated with AIIB2 nanowires followed by GSH exchange had significantly more linear junctions, as based on the junction length ratio, which was comparable to that of untreated cells or cells treated with bare nanowires (Fig. 3E). Taken together, these results show that the ability of AIIB2 nanowires to cause tight junction ruffling can persist for an extended period of time, is reversible and is dependent on the persistent engagement of multivalent antibody-conjugated nanowires.

### Effect of anti-integrin nanowires on claudins and adherens junctions

**Fig. 4.**
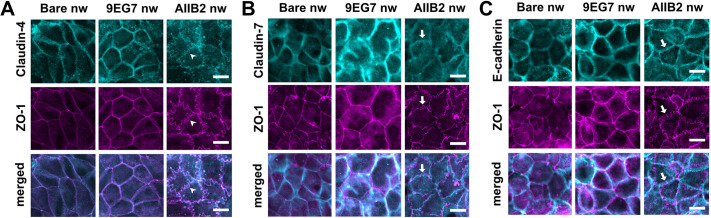
**Nanowires differentially affect different claudins.** (A–C) Representative immunofluorescence images of Caco-2 cells colabeled to detect ZO-1 (magenta) and either claudin-4 (A), claudin-7 (B) or E-cadherin (C) (all cyan). Cells were fixed 2 h after treatment with either bare, 9EG7 or AIIB2 nanowires (nw). Arrowheads show the ruffled appearance of claudin-4 colocalizing with ZO-1; arrows indicate areas of claudin-7 (B) and E-cadherin (C) that do not colocalize with ruffled ZO-1. Scale bars: 10 μm. Images are representative of three independent experiments.

Despite robust evidence suggesting that a variety of stimuli can induce ruffling of tight junctions based on ZO-1 staining, fewer studies have examined how claudins are affected when ZO-1 is ruffled (Lynn et al., 2020). We thus examined the impact of anti-integrin nanowires on claudins expressed by Caco-2 cells to determine whether this paralleled the changes in ZO-1 morphology. Stimulation of cells with AIIB2 nanowires caused claudin-4 (Fig. 4A) and claudin-2 (Fig. S4B) to acquire a ruffled morphology, where both claudins colocalized with ruffled ZO-1. By contrast with claudin-2 and claudin-4, we observed that AIIB2 nanowires did not cause tight junction-localized claudin-7 (Fig. 4B) and claudin-1 (Fig. S4A) to adopt a ruffled morphology (Walsh et al., 2015). In contrast to the effects of treatment with AIIB2 nanowires, treatment with 9EG7 nanowires did not induce claudin ruffling. Instead, 9EG7 nanowires caused claudin-2 and claudin-4 to adopt a morphology with increased linearity (Fig. 4A; Fig. S4B). Claudin-1 and claudin-7 also were not ruffled in response to treatment with 9EG7 nanowires. Instead, claudin-1 and claudin-7 largely colocalized with ZO-1; however, there also were pools of claudin-1 and claudin-7 that did not colocalize with ZO-1 in cells treated with 9EG7 nanowires (Fig. 4B; Fig. S4A).

We then measured the effect of anti-integrin nanowires on the adherens junction proteins E-cadherin (CDH1) (Fig. 4C) and β-catenin (CTNNB1) (Fig. S4C). None of the treatments caused the localization of either E-cadherin or β-catenin to become ruffled. However, treatment of cells with AIIB2 nanowires resulted in a β-catenin localization that was less intense and more dispersed than that in cells treated with bare nanowires. Treatment with 9EG7 nanowires also led to reduced β-catenin immunofluorescence intensity but had less impact on the junctional localization of β-catenin, and although there were areas where β-catenin was dispersed, this was to a lesser extent than seen in cells treated with AIIB2 nanowires.

We also determined the impact of nanowires on total claudin-4, claudin-7 and E-cadherin by measuring immunofluorescence images for the intensity of junction-associated protein (Fig. S4D). In each case, we found that there was not a significant difference in intensity, indicating that the predominant effect of nanowires on these transmembrane junctional proteins was to alter their localization, rather than total expression.

### Effect of anti-integrin nanowires on barrier function

**Fig. 5.**
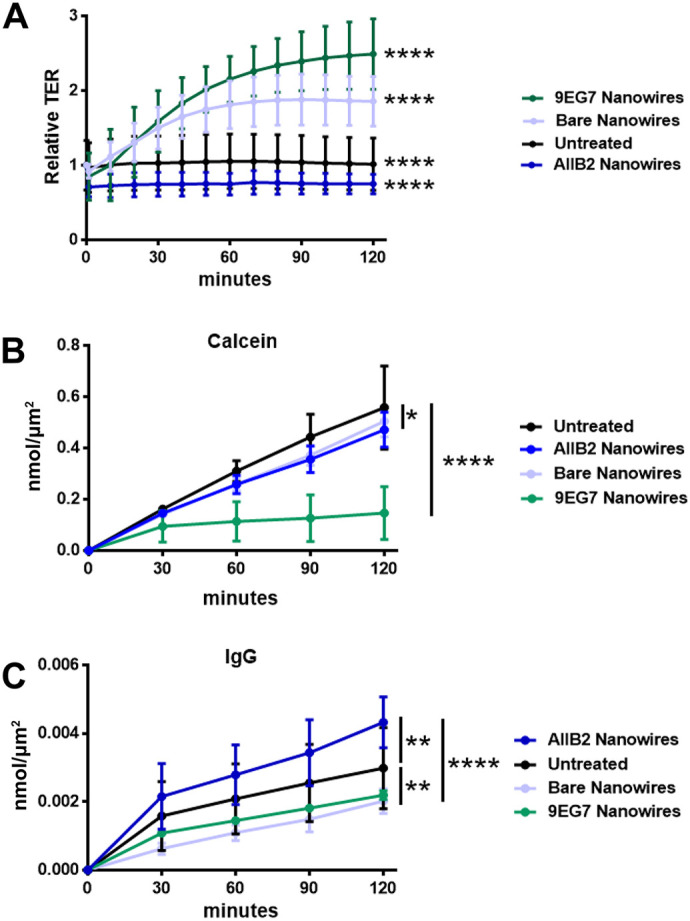
**Nanowires alter epithelial barrier function.** (A) TER measurements on Caco-2 cells for 2 h following treatment as indicated. Measurements were taken every 10 min with a cellZscope impedance system, and all points were normalized to baseline TER readings before treatment. Each point is the average TER for that treatment±s.d. (*n* wells/treatment: AIIB2, *n*=9; 9EG7, *n*=7; bare, *n*=6; untreated, *n*=10). ****P<0.0001 (all treatments were significantly different from the others). (B) Dye flux permeability, as assayed using calcein (630 Da), for Caco-2 cells with the indicated treatments. Each point is the average permeability for that treatment±s.d. (*n* wells/treatment: AIIB2, *n*=7; 9EG7, *n*=8; bare, *n*=7; untreated, *n*=5). *P=0.017 (untreated versus AIIB2 nanowires), ****P<0.0001 (untreated versus 9EG7 nanowires). (C) Dye flux permeability, as assayed using whole fluorescently tagged IgG (160 kDa), for Caco-2 cells with the indicated treatments. Each point is the average permeability for that treatment±s.d. (*n* wells/treatment: AIIB2, *n*=6; 9EG7, *n*=4; bare, *n*=4; untreated, *n*=6). **P=0.016 (untreated versus AIIB2 nanowires), **P=0.013 (untreated versus bare nanowires), ****P<0.0001 (AIIB2 nanowires versus bare nanowires and 9EG7 nanowires). In each case, significance was determined by two-way ANOVA with Tukey correction for multiple comparisons.

We have previously demonstrated that when cells are treated with a nanostructured surface that causes a ruffled ZO-1 morphology, the ruffling is accompanied by decreases in barrier function (Kam et al., 2013; Stewart et al., 2017; Walsh et al., 2015). Thus, we examined the effect of anti-integrin nanowires on Caco-2 cell barrier function. Initially, we measured TER for the duration of a 120 min time course of treatment (Fig. 5A). Relative to bare nanowires, AIIB2 nanowires and 9EG7 nanowires had differential effects on TER, where AIIB2 nanowires caused a decrease in TER whereas 9EG7 nanowires increased TER greater than twofold over 120 min. Thus, nanowires targeting integrin β1 in an inactive, bent conformation caused increased paracellular ion permeability, whereas nanowires targeting active integrin β1 had the opposite effect and promoted barrier function. Bare nanowires also significantly increased TER, although to a lesser extent than 9EG7 nanowires.

We then examined the effects of anti-integrin nanowires on paracellular flux, using calcein (630 Da; Fig. 5B) as a small-molecule tracer. Treatment of cells with AIIB2 nanowires had no significant effect on the paracellular permeability of calcein, as compared with that of cells treated with bare nanowires; however, when compared with untreated cells, cells treated with AIIB2 nanowires were found to exhibit a small decrease in calcein flux. By contrast, treatment with 9EG7 nanowires significantly decreased calcein flux as compared with the other treatments examined. Thus, the effects of 9EG7 nanowires on paracellular flux of calcein paralleled their effects on TER.

The impact of anti-integrin nanowires on transcytosis was also examined, using fluorescently tagged IgG, which is minimally transported through the paracellular route (Stewart et al., 2017). Cells treated with AIIB2 nanowires displayed significantly enhanced IgG transport as compared with that of untreated cells or cells treated with bare or 9EG7 nanowires (Fig. 5C). By contrast, bare and 9EG7 nanowires significantly decreased IgG transport as compared with that in untreated cells. Taken together, these data demonstrate that different modes of targeting integrins have different effects on Caco-2 barrier function. Whether the ability of bare PCL nanowires to enhance barrier function is integrin-dependent remains to be determined; however, there is precedent for non-proteinaceous substrates to specifically stimulate integrins (Huang et al., 2020; Lamson et al., 2020).

### Effect of targeting apical integrin β1 subunits on talin and actin

**Fig. 6.**
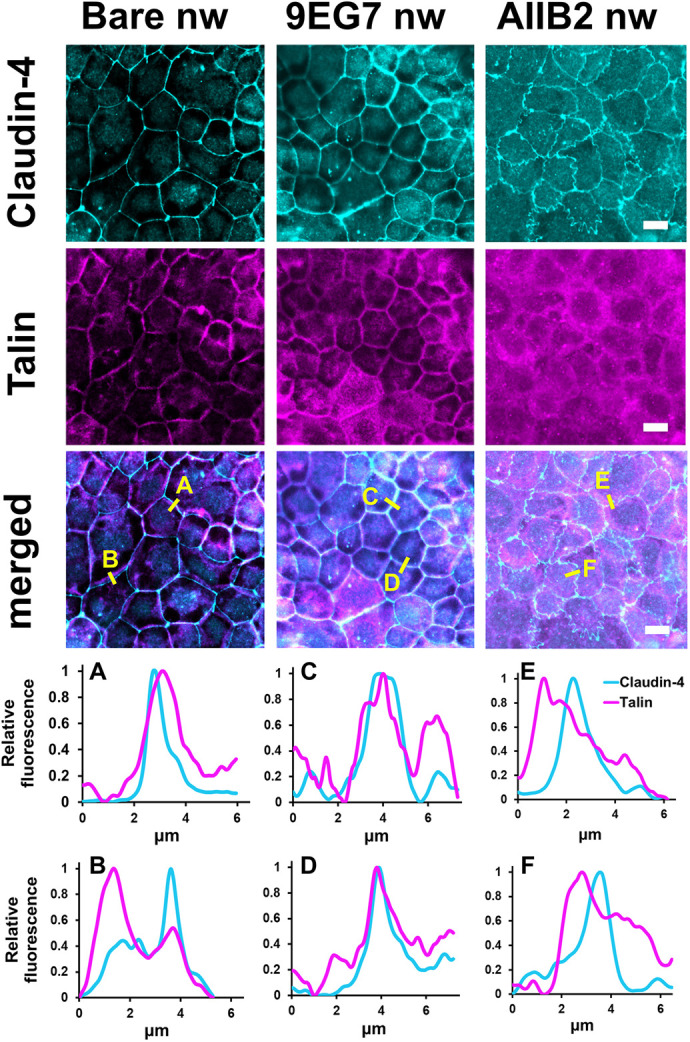
**Talin reorganization in response to nanowires.** Representative immunofluorescence images of talin (magenta) and claudin-4 (cyan) in Caco-2 cells fixed 2 h after the indicated nanowire (nw) treatment. Representative line scans as indicated in the merged images were measured using ImageJ to indicate colocalization of talin and claudin-4 in cells treated with bare nanowires (A,B), 9EG7 nanowires (C,D) or AIIB2 nanowires (E,F). Cells treated with bare nanowires or 9EG7 nanowires showed good concordance between talin and claudin-4; however, the distribution of junction-associated talin did not match the distribution of claudin-4 in cells treated with AIIB2 nanowires. Scale bars: 10 μm. Images are representative of two independent experiments.

There are several lines of evidence supporting the role of apically localized integrins in the regulation of tight junction structure and barrier function, but little has been done to determine what links apical integrins to regulation of the epithelial barrier. Talins are scaffold proteins that directly bind the cytoplasmic tail of integrin β1 to function as an actin crosslinker (Burridge and Connell, 1983; Ciobanasu et al., 2013; Horwitz et al., 1986). Talin-1 has recently been found to have an important role in regulating vascular barrier function (Pulous et al., 2019). Thus, we examined the effect of nanowires on talin localization using a pan-talin antibody. Unless otherwise noted, talin herein refers to talin-1 and talin-2. In cells treated with bare or 9EG7 nanowires, there was concordant overlap between talin and claudin-4, which was used as a marker for tight junctions (Fig. 6). On the other hand, the distribution of talin did not match that of claudin-4 in cells treated with AIIB2 nanowires. Instead, in AIIB2 nanowire-treated cells, areas containing ruffled claudin-4 tended to be associated with more disorganized pools of talin.

**Fig. 7.**
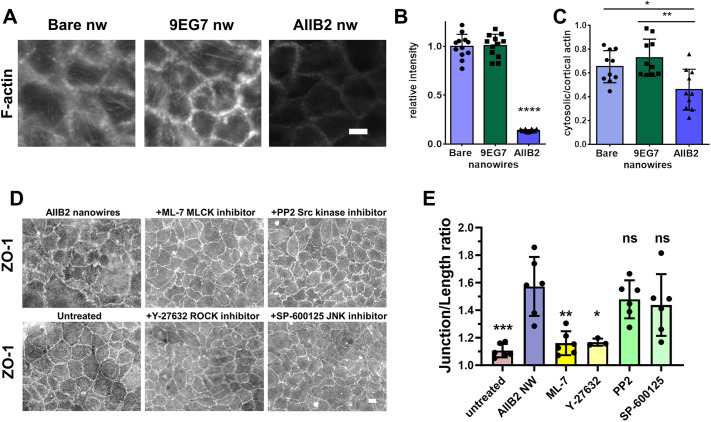
**Actin reorganization in response to nanowires.** (A) Representative fluorescence images of F-actin (labeled using rhodamine–phalloidin) in Caco-2 cells fixed 2 h after the indicated nanowire (nw) treatment. Scale bar: 10 μm. (B) Fluorescence intensity measurements of cells as in A. Data are presented as mean±s.d. (*n*=4 fields of view from three slides per condition). ****P<0.0001 for AIIB2 nanowire-treated cells compared with cells treated with either bare nanowires or 9EG7 nanowires. (C) The ratio of cytosolic (stress fibers) to cortical actin for cells as in A. Data are presented as mean±s.d. calculated from 10 cells in a representative field, showing less cytosolic actin for AIIB2 nanowire-treated cells. **P=0.0018, *P=0.025. Significance determined by one-way ANOVA with Bonferroni correction for multiple comparisons. (D) Caco-2 cells were pre-incubated with either ML-7 (10 μM), Y-27632 (10 μM), PP2 (10 μM), SP-600125 (1 μM) or vehicle control for 30 min followed by 2 h treatment with AIIB2 nanowires, except for the untreated group, which was not exposed to nanowires or inhibitors. Cells were then fixed, immunostained for ZO-1, and imaged. Representative images of ZO-1 immunostaining are shown. Scale bar: 10 μm. (E) Junction length ratios were calculated for cells as in D. Data are presented as mean±s.d. [*n*=3 fields of view from one slide (Y-27632) or two slides per condition; 15 measurements per field of view]. ML-7 and Y-27632 significantly inhibited the effects of A2BII nanowires on junction length ratio (**P=0.0011; *P=0.013). Untreated cells had significantly lower junction length ratio than control AIIB2 nanowire-treated cells (***P<0.0002). Significance was determined by one-way ANOVA with Bonferroni correction for multiple comparisons (ns, not significant).

Since actin has been implicated in the ability of nanostructured films to induce tight junction ruffling (Walsh et al., 2015), we hypothesized that integrin-induced changes in actin cytoskeleton organization might be responsible for the observed cell responses to anti-integrin nanowires. Using rhodamine–phalloidin to stain for F-actin revealed that treatment of cells with AIIB2 nanowires caused an overall decrease in total F-actin as compared to F-actin levels in cells treated with either bare or 9EG7 nanowires (Fig. 7A–C). In addition, cells treated with AIIB2 nanowires lacked actin stress fibers. By contrast, there was a pool of cortical (junction-associated) actin in the cells regardless of nanowire treatment. Actin rearrangement was confirmed by quantitation, comparing the ratio of the intensity of cytosolic (stress fiber-associated) actin to that of cortical actin (Fig. 7C).

To identify roles for kinases that regulate the actin cytoskeleton and tight junctions in nanowire-induced tight junction ruffling, cells were pre-incubated for 30 min with either ML-7, an inhibitor of myosin light chain kinases (MLCKs; Turner et al., 1997); Y-27632, an inhibitor of Rho kinases (ROCKs; Walsh et al., 2001); PP2, an inhibitor of Src kinase (Sanders and Basson, 2004); SP-600125, an inhibitor of Jun kinases (JNKs; Samak et al., 2011); or vehicle control. Pre-incubation was followed by a 2 h incubation with AIIB2 nanowires and then immunofluorescence analysis for ZO-1 morphology (Fig. 7D,E). Of these inhibitors, only ML-7 and Y-27632 inhibited the formation of tight junction ruffles in response to AIIB2 nanowires. This is consistent with multiple studies demonstrating that inhibition of MLCKs or ROCKs has the capacity to strengthen barrier function (Citi et al., 2024; He et al., 2020; Ivanov et al., 2010).

**Fig. 8.**
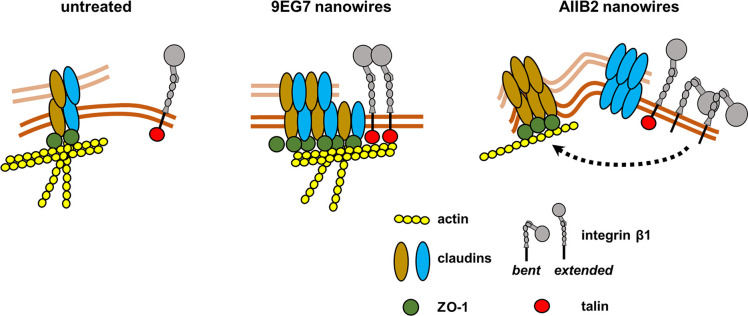
**Model of the impact of anti-integrin nanowires on organization of tight junction proteins and actin.** In this model, 9EG7 activating nanowires cluster integrin β1 to promote recruitment of actin, ZO-1 and talin to sites where tight junctions form, increasing their function. By contrast, AIIB2 blocking nanowires decrease actin and talin recruitment, which causes claudins to separate into distinct pools, increasing tight junction permeability.

Taken together, these data support a model where 9EG7 nanowires promote barrier function by inducing a complex between tight junction proteins, actin and talin, whereas AIIB2 nanowires induce tight junction ruffling by disconnecting tight junction proteins from cortical actin and associated regulatory proteins, including talin (Fig. 8).

## DISCUSSION

In this study we have demonstrated that specifically targeting apically localized integrin β1 is sufficient to induce changes in tight junction morphology and barrier function. Furthermore, for the first time, we have found that targeting different conformations of apical integrin β1 subunits with antibodies results in differential regulation of epithelial barrier structure and function. In other words, integrin β1 can act as a switch to either increase or decrease epithelial barrier function, depending on the stimulus.

We found that nanowires decorated with an anti-integrin β1 antibody classically considered to be blocking (AIIB2) caused an increase in paracellular ion permeability, based on TER. AIIB2 nanowires also enhanced IgG transcytosis; however, there was little effect of AIIB2 nanowires on paracellular flux of solutes. Moreover, these responses required a multivalent substrate, since monovalent AIIB2 did not cause tight junction ruffling. The requirement for a multivalent substrate was also demonstrated by GSH treatment of cells stimulated by AIIB2 nanowires – although the AIIB2 antibodies were likely to remain engaged to integrins at the cell surface following GSH treatment, release of the nanowires was found to reverse the effect on tight junctions. Moreover, Caco-2 cell responses were not limited to AIIB2 antibodies, since nanowires decorated with another blocking antibody, mAb13, also induced tight junction ruffling.

The classification of AIIB2 and mAb13 as blocking antibodies reflects their ability to inhibit processes that require active integrins (Hall et al., 1990; Spiess et al., 2018; Werb et al., 1989). Our data demonstrate that, rather than being inert, apically localized integrin β1 in a closed state can influence cell function when engaged in a specific manner, such as being clustered by a multivalent ligand and a substrate with a high aspect ratio (e.g. rod-shaped versus spherical). Taken together, our data suggest that multivalent delivery and persistent nanostructured engagement are requirements for apical integrins to be activated to increase barrier permeability, suggesting a mechanism of action for barrier regulation via clustering of inactive integrins (Burridge and Chrzanowska-Wodnicka, 1996). Consistent with this model, pools of inactive integrin β1 have been shown to be localized to intercellular junctions (Yanez-Mo et al., 2001) and in subdomains of focal adhesions (Spiess et al., 2018), which might have functional significance.

The effects of AIIB2 nanowires on tight junction morphology (ruffled), TER (decreased), paracellular flux (little effect) and transcytosis (increased) match Caco-2 cell responses to nanostructured films placed in contact with the apical surface (Huang et al., 2020; Kam et al., 2013; Stewart et al., 2017; Walsh et al., 2015). Integrins have been shown to play a role in the ability of nanostructured films to alter epithelial barrier function (Walsh et al., 2015), as demonstrated by two key experiments: (1) pre-incubation of cells with monomeric integrin blocking antibodies prior to film engagement inhibits the effect of nanotopography on barrier function, and (2) pre-incubation of cells with RGD peptides prior to film engagement enhances the effect of the substrate on barrier function. The integrin blocking experiment is consistent with the results we obtained where free AIIB2 antibody had little effect on epithelial cells as compared with AIIB2 nanowires.

The observation that RGD peptide stimulation amplifies the effects of nanostructured films on cells is less straightforward to interpret. Previous work has shown that soluble RGD peptide concentrations as high as 5 mg/ml do not alter Caco-2 cell barrier function (Cruz et al., 1994). Since RGD peptides primarily activate the integrin β1 pool that is heterodimerized with integrin αv, integrin α5 or integrin α8 (Hynes, 2002), the inability of RGD peptides to alter barrier function suggests that tight junctions are regulated by other integrins expressed by Caco-2 cells, such as heterodimers recognizing collagen (e.g. α2β1) or laminin (e.g. α6β1) (Basson et al., 2000). In contrast to ligands, anti-integrin β1 antibodies have the potential to interact with all integrin β1 heterodimers (Byron et al., 2009), irrespective of the α subunit. Thus, in the context of nanostructured films, RGD pre-treatment might facilitate engagement of other integrin β1 pools with nanostructured films, resulting in altered barrier function. Whether this is the case remains to be determined.

Ruffling of tight junctions has also been observed in Caco-2 cells mechanically stimulated by cyclic stretch (Samak et al., 2014). In that study, inhibitors were used to determine that MLCKs, c-Src and JNKs are all required for stretch to cause tight junction ruffling. Given this, we tested several agents for the ability to inhibit AIIB2 nanowires from inducing tight junction ruffling. We found that ML-7 and Y-27632 inhibited ruffling, implicating roles for MLCKs and ROCKs in the response to AIIB2 nanowires. MLCKs have also been found to be required for ruffling and decreased barrier function in Caco-2 cells stimulated with nanostructured films (Walsh et al., 2015), suggesting a common mechanism shared between AIIB2 nanowires and nanostructured films. On the other hand, PP2 and SP-600125 had no effect, indicating that c-Src and JNKs were not required for AIIB2 nanowire-induced ruffling, in contrast to stretch-induced ruffling, suggesting that there are multiple pathways that can lead to ruffled tight junctions. For instance, depletion of p21-activated kinase 2 (PAK2) (Tornavaca et al., 2015) decreases claudin-1 expression (Saeedi et al., 2015), and overexpression of dominant-negative RhoA and Rac1 constructs (Jou et al., 1998) has been shown to induce ZO-1 to adopt a ruffled morphology.

Our data also support a model where AIIB2 nanowires cause a decrease in TER by sorting claudins into two distinct pools, defined by claudins that colocalize with ruffled ZO-1 (claudin-2 and claudin-4) and those that do not (claudin-1 and claudin-7). Sorting claudins into at least two classes of tight junction strands with different claudin composition is a direct means to alter paracellular permeability (Capaldo et al., 2014). Also, it has been proposed that tight junction ruffling can increase paracellular ion permeability by increasing tight junction length, thereby increasing their capacity for ion-permeable claudins (such as claudin-2) as compared with linear tight junctions (Gonzalez-Mariscal et al., 2001).

Although claudin–ZO-1 interactions have been implicated in the appearance of ruffled tight junctions (Lynn et al., 2020), to our knowledge no work to date has shown whether claudins themselves are recruited into ruffles. Previous work has primarily demonstrated a link between ruffled ZO-1 and changes in claudin expression, rather than claudin morphology. Ruffled ZO-1 resulting from various stimuli coincides with decreases in expression of claudin-1 (Saeedi et al., 2015; Tokuda et al., 2014; Walsh et al., 2015), increases in expression of claudin-2 and decreases in expression of claudin-7 (Tokuda et al., 2014). Interestingly, different stimuli inducing tight junction ruffling can result in either increased (Jin and Blikslager, 2016) or decreased (Walsh et al., 2015) claudin-4 expression. Moreover, claudin-2 and claudin-4 are known to interact and compete for localization to tight junctions (Capaldo et al., 2014), which is consistent with their ability to localize into ruffled junctions. On the other hand, claudin-1 and claudin-7, which are phylogenetically similar based on their C-terminal tails (Krause et al., 2008), were not associated with ruffled ZO-1. These data suggest that claudin localization to ruffles is controlled by specific molecular motifs and is likely comparable to structural elements that determine heteromeric compatibility (Gonschior et al., 2022).

In contrast to AIIB2 nanowires, 9EG7 nanowires were associated with improved barrier function, as noted by an increase in TER, a decrease in paracellular flux, a decrease in transcytosis and hyper-linearized junctions. Nanowires decorated with another activating antibody, 12G10, also caused tight junctions to hyper linearize, in support of integrin activation enhancing epithelial barrier function. Consistent with this model, a vitronectin–integrin axis has recently been found to regulate blood–brain barrier function, where activated integrins inhibit transcytosis (Ayloo et al., 2022; Trevino and Lutz, 2022).

Cells treated with 9EG7 nanowires also showed an enhancement of talin colocalization with tight junctions. Talin-1 has been implicated in promoting barrier function (Pulous et al., 2019), and talin-1 enrichment is also observed when the apical surfaces of cells are exposed to integrin-stimulating substrates (Huang et al., 2020). Taken together, these observations suggest that recruitment of talin to tight junctions is part of the mechanism of action for the ability of 9EG7 nanowires to increase epithelial barrier function. A potential role for talin in this context is to promote interactions between tight junctions and actin. Whether tight junction permeability is specifically controlled by talin-1 or is also regulated by talin-2 remains to be determined. This could involve formation of a complex between integrins, tight junction proteins and scaffold proteins. Consistent with this possibility, claudin-7 has been shown to directly interact with integrin β1 (Ding et al., 2016; Kim et al., 2019; Lu et al., 2015). The barrier-forming protein junctional adhesion protein A (JAM-A, also known as F11R) has also been found to associate with integrins (Kummer et al., 2022; Tholmann et al., 2022). However, to date, integrin association with either claudin-7 or JAM-A have been associated with cell functions such as proliferation and migration, so it remains an open question as to whether integrin association with these proteins and potentially other tight junction proteins can regulate barrier function.

By contrast, cells treated with AIIB2 nanowires showed a decrease in actin and less talin associated with tight junctions. The resultant loss of cortical actin and junction-associated talin are expected to have a destabilizing effect, allowing tight junctions to assume a ruffled morphology and leading to increased permeability.

A practical implication of our findings is that anti-integrin nanowires might have therapeutic relevance, either as agents to promote barrier permeability to improve drug delivery (Walsh et al., 2015) or to promote barrier function of epithelia that are pathologically leaky (Den Beste et al., 2013). PCL is a good substrate for these applications, since it is biodegradable and the lifetime can be controlled by altering the molecular mass of the PCL monomers used to form the polymer (Bartnikowski et al., 2019). PCL also has the capacity for incorporation of pharmacologic agents that could improve efficacy (Dash and Konkimalla, 2012), such as growth factors (Finbloom et al., 2020). Our results suggest that the combination of vehicle composition and geometry coupled with conformation-specific ligands targeting integrins will enable the design of targeted therapeutic agents.

## MATERIALS AND METHODS

### Fabrication and conjugation of nanowires

Derivitizable polycaprolactone (PCL) nanowires were fabricated from a mix of 45 kDa PCL (Sigma Aldrich, 704105) and maleimidophenyl-PCL (MP-PCL) synthesized as described previously, with modifications (Zamecnik et al., 2020, 2017). Briefly, the PCL mixture (total polymer concentration 125 mg/ml with MP-PCL as 30% of total polymer weight) was added to 2,2,2-trifluorethanol (Sigma Aldrich, T63002) before being spin coated onto glass slides (Fisherbrand, 12-550C) in two stages: 500 rpm (11 ***g***) for 10 s, followed by 1000 rpm (45 ***g***) for 30 s using a Ni-Lo 5 Vacuum Holder Digital Spin Coater (Ni-Lo Scientific, Ottawa, ON, Canada). In some experiments, Nile Red (mass ratio of 125:1 PCL:Nile Red) was added to the PCL mixture to produce fluorescent nanowires for imaging. Anodized aluminium oxide (AAO) anapore wafers with a 200 nm pore served as the template for the nanowires (Sigma Aldrich, WHA68095502) and were placed in contact with the polymer film before heating the film to 100°C for 3 h to complete the templating process before cooling overnight. The wafers were then removed from the slide and etched in 5 M NaOH for 30 min at 4°C. The etchant was passed through a 0.22 µm PES filter (Corning, 431118) and rinsed first with cold distilled water, followed by a rinse with cold PBS (Corning, 21-040-CV). Nanowires were removed from the filter by rinsing with a 5% (v/v) solution of poly(vinyl alcohol) (PVA; Sigma Aldrich, 475904) in water before being passed through 40 µm mesh (Corning, 352340). The filtered nanowires were centrifuged (Eppendorf, 5810R) three times at 4000 rpm (3220 ***g***) for 15 min at 4°C, the supernatant was discarded, and the pelleted nanowires were washed sequentially with cold distilled water, with cold PBS and with reducing buffer (PBS with 0.04% w/v EDTA), and were then stored in reducing buffer at 4°C until use.

Nanowires were conjugated with either AIIB2 blocking anti-integrin β1 antibody (Millipore, MABT409), mAb13 blocking anti-integrin β1 antibody (Millipore, MABT821), 9EG7 activating anti-integrin β1 antibody (BD Pharmingen, 553715), 12G10 activating anti-integrin β1 antibody (Millipore, MAB2247) or an isotype control antibody (Thermo Fisher Scientific, 31933).

To conjugate antibodies with the nanowires, the antibodies were first diluted to 0.2 mg/ml before being reduced with tris(2-carboxyethyl)phosphine (TCEP; Sigma Aldrich, 646547) in reducing buffer at a 4.5 molar excess for 1 h at 37°C. An equal volume of nanowires was added to the reduced antibodies where the thiol-maleimide reaction proceeded for 2 h at 25°C. Using this approach, nanowires are conjugated to antibody at a mass ratio of 50:1 (50 µg nanowires to 1 µg antibody). Conjugated nanowires were washed three times and centrifuged (Eppendorf, 5810R) at 2500 rpm (1258 ***g***) for 10 min at 4°C, discarding the supernatant and resuspending pellets in fresh PBS. Antibody-conjugated nanowires were either used within 4 h of conjugation or stored at 4°C, which enabled them to be used within 36 h of conjugation.

Microspheres were fabricated using a single-emulsion technique by adding the PCL/MP-PCL mixture, as described above, dropwise into a 1.5% PVA solution in water. The emulsion was then sonicated on ice in 10 s bursts for 1 min before being added to excess PVA mixing on a magnetic stir plate. Microspheres were centrifuged (Eppendorf, 5810R) at 1400 rpm (400 ***g***) for 15 min at 4°C, collected, washed three times with cold PBS, resuspended in reducing buffer, filtered through 40 μm mesh (Corning, 352340) and then stored in reducing buffer at 4°C until use. Antibodies were conjugated onto microspheres in a manner comparable to nanowire conjugation.

### Cell culture

Caco-2 cells (ATCC, HTB-37) were maintained in Minimum Essential Medium (MEM) with Earle's salts and L-glutamine (Corning Cellgro, 10-010-CV) supplemented with 10% fetal bovine serum (Atlanta Biologicals Premium Select, S11550), sodium pyruvate (Hyclone, SH30239.01), 100 U/ml penicillin and 10 mg/ml streptomycin (Sigma Aldrich, P4333), 0.25 µg/ml amphotericin B (Thermo Fisher Scientific, 15290018), and 5 µg/ml gentamicin (Sigma Aldrich, G1397). Cells were incubated in a CO_2_ incubator at 37°C until they were ready to be seeded to glass coverslips for immunofluorescence experiments or Transwell permeable supports for barrier function experiments.

### Immunofluorescence and image analysis

Caco-2 cells were seeded at 200,000 cells per well on rat-tail collagen (Roche, 11179179001)-coated coverslips in the supplemented MEM medium as described above. Cells were incubated at 37°C for ∼4 days until they reached confluency. Unless otherwise stated, cells were treated with nanowires and incubated for 2 h in a CO_2_ incubator at 37°C before being prepared for immunofluorescence. For 6.5 mm wells, the net amount of antibody added for each treatment was 0.612 µg/well. Free reduced antibodies were produced with TCEP as described above, but in the absence of nanowires, and were added directly to cell cultures to a final concentration of 0.612 μg/well. In some experiments, cells were pretreated for 30 min at 37°C with medium containing an inhibitor (added from a 1000× stock) or vehicle control prior to addition of nanowires. Inhibitors tested were ML-7 (Abcam, ab120848-10MG), Y-27632 (Selleck Chemicals, S1049-10MG), PP2 (Abcam, ab120308-10MG) and SP-600125 (Abcam, ab120065-50MG).

After the incubation period, the cells were washed in PBS with calcium and magnesium (Corning, 21-030-CV), then fixed with 4% paraformaldehyde (PFA) (Electron Microscopy Solutions, 15710). In most preparations, PFA fixation was followed by a methanol:acetone fixation step (methanol: Fisher Chemical, A433F; acetone: Fisher Chemical, A19-1) for 2 min at room temperature (Table S1). The cells were then permeabilized with 0.5% Triton X-100 (Fisher Scientific, 9002-93-1). Primary antibody (Table S1) was diluted in 3% bovine serum albumin (BSA; Gemini Bio-Products, 700-102P) and incubated on cells overnight at 4°C. Cells were washed with 3% BSA before Alexa Fluor 594-conjugated goat anti-rabbit IgG (Thermo Fisher Scientific, A32740) and Cy2-conjugated goat anti-mouse IgG (Jackson ImmunoResearch Labs, 115-225-166) secondary antibodies were diluted in 3% BSA and incubated on cells for 1 h at room temperature. Samples were then washed with PBS with calcium and magnesium before the coverslips were mounted on slides with ProLong antifade containing DAPI (Thermo Fisher Scientific, P36962). For F-actin visualization, rhodamine–phalloidin (Thermo Fisher Scientific, R415) was diluted in PBS and incubated with cells overnight at 4°C before mounting the coverslips.

Images were acquired using an Olympus IX70 microscope with a U-MWIBA filter pack (BP460-490, DM5050, BA515-550) or U-MNG filter pack (BP530-550, DM570, BA590-800) and with a UPlanFl 60× 1.25 oil iris objective, or a Nikon A1R HD25 confocal microscope with an N 60× 1.40 Oil Apo Lambda S DIC N2 objective. Sample identification was covered on slides before imaging to minimize bias in image collection. All images were processed in ImageJ (Schneider et al., 2012), each image had background subtracted with a rolling ball radius of 50 pixels. Minimum and maximum intensities for images of the same protein were adjusted in parallel so the intensity scale remained linear.

Image quantification was done with the sample identity hidden using ImageJ. The junction length ratio (A/B) between two tricellular junctions (Higashi and Chiba, 2020) was determined using the freehand line tool to manually trace tight junctions, providing the actual junction length (A), and the straight line tool to measure the minimum linear distance (B). Percent of cells with ruffled junctions was determined by counting the number of cells with at least one ruffled junction (junction length ratio>1.3) normalized to the total number of cells in a given field. Line scans of double-labeled fluorescence images were made using the straight line tool to define a region of interest (ROI) across a tight junction followed by measuring the fluorescence intensity of each fluorophore using the plot profile function of ImageJ. Intensity profiles were individually background corrected and normalized to the maximum intensity measured along the line scan.

### Barrier function assays

Caco-2 cells were seeded at 250,000 cells per well in the apical chamber of a 6.5 mm permeable support (i.e. Transwell) from either Corning (3470) or CellTreat (230635). Both brands of inserts gave comparable results. Cells were grown with 200 µl of the supplemented MEM medium described above in the apical chamber (permeable support) and 1 ml of the supplemented MEM medium in the basolateral chamber. Cells were incubated at 37°C and medium was changed every other day for ∼7 days until cells formed a high-resistance monolayer of 350 Ω.cm^2^ or higher. Monolayer resistance was measured using an epithelial voltohmmeter (World Precision Instruments, Sarasota, FL, USA) where the measured resistance in ohms was multiplied by the area of the Transwell filter (0.33 cm^2^).

To ensure that changes in barrier function were not the result of cell death, we measured cell viability (Fig. S1) with a colorimetric live–dead assay (Thermo Fisher Scientific, L3224). Cells seeded in Transwells were treated for 2 h with nanowires, soluble antibody or antibody-conjugated nanowires. After experimental treatment, cells were incubated with 4 µM of ethidium homodimer-1, 2 µM calcein-AM, and Hoechst 33342 (Thermo Fisher Scientific, H1399) for 30 min at room temperature. Cells were directly imaged through the Transwell on a glass-bottomed 35 mm dish (MatTek, P35G-1.0-14-C), and percent viability was calculated by scoring the percentage of calcein-positive (live) and ethidium-positive (dead) cells in each field of view.

Transepithelial electrical resistance (TER) was measured over a time course of 2 h using the cellZscope 2 and its accompanying software for data acquisition (nanoAnalytics, Münster, Germany). Data were pooled from 5–10 wells per treatment, where each well was normalized to a baseline TER measurement before treatment. The averages of the normalized TER for each treatment were plotted over the 2 h time course. Significance between conditions was determined by doing a one-way repeated measures ANOVA with multiple comparisons and Bonferroni correction (GraphPad Prism, Dotmatics, San Diego, CA, USA).

Permeability for larger molecules was assessed using a dye flux assay where high-resistance monolayers of Caco-2 cells seeded on 6.5 mm Transwell inserts as above were equilibrated with Ringer's solution (140 mM NaCl, 2 mM CaCl_2_, 1 mM MgCl_2_, 10 mM glucose and 10 mM HEPES pH 7.3) for 30 min at 37°C before the apical buffer was replaced with fresh Ringer's solution containing either 2 μg/ml calcein (Thermo Fisher Scientific, C481) or 8.3 μg/ml Alexa Fluor 488-labeled IgG (JacksonImmuno Research Labs, 711-545-152). Over a 2 h time course, Transwell inserts were moved to new wells containing 200 µl Ringer's solution every 30 min. After Transwell inserts were moved, the Ringer's solution in the basolateral chamber was collected to be read using a multichannel plate fluorimeter (BioTek-Synergy H Microplate Reader, Winooski, VT, USA). Data were pooled from 4–6 wells per treatment, and using a standard curve, absolute flux was calculated. Significance between conditions was determined two-way ANOVA with multiple corrections to compare simple row effects between time points and Bonferroni correction (GraphPad Prism).

### Glutathione competition

A 20 mM solution of L-glutathione reduced (Sigma Aldrich, G6013) solubilized in deionized water was diluted to 1 mM in fresh supplemented MEM medium (as described above) before being added to anti-integrin nanowire-treated coverslips and incubated for 8 h. After incubation, cells were then prepared for immunofluorescence.

## Supplementary Material

10.1242/joces.263580_sup1 Supplementary information

## References

[JCS263580C1] Ayloo, S., Lazo, C. G., Sun, S., Zhang, W., Cui, B. and Gu, C. (2022). Pericyte-to-endothelial cell signaling via vitronectin-integrin regulates blood-CNS barrier. *Neuron* 110, 1641-1655.e6. 10.1016/j.neuron.2022.02.017 35294899 PMC9119930

[JCS263580C2] Baldwin, A. D. and Kiick, K. L. (2011). Tunable degradation of maleimide-thiol adducts in reducing environments. *Bioconjug. Chem.* 22, 1946-1953. 10.1021/bc200148v 21863904 PMC3220410

[JCS263580C3] Bartnikowski, M., Dargaville, T. R., Ivanovski, S. and Hutmacher, D. W. (2019). Degradation mechanisms of polycaprolactone in the context of chemistry, geometry and environment. *Prog. Polym. Sci.* 96, 1-20. 10.1016/j.progpolymsci.2019.05.004

[JCS263580C4] Basson, M. D., Emenaker, N. J. and Sanders, M. A. (2000). Alpha integrin subunits regulate human (Caco-2) intestinal epithelial proliferation and phenotype. *Cell. Physiol. Biochem.* 10, 27-36. 10.1159/000016332 10844395

[JCS263580C5] Bazzoni, G., Shih, D. T., Buck, C. A. and Hemler, M. E. (1995). Monoclonal antibody 9EG7 defines a novel beta 1 integrin epitope induced by soluble ligand and manganese, but inhibited by calcium. *J. Biol. Chem.* 270, 25570-25577. 10.1074/jbc.270.43.25570 7592728

[JCS263580C6] Buckley, A. and Turner, J. R. (2018). Cell biology of tight junction barrier regulation and mucosal disease. *Cold Spring Harb. Perspect. Biol.* 10, a029314. 10.1101/cshperspect.a029314 28507021 PMC5749156

[JCS263580C7] Burridge, K. and Chrzanowska-Wodnicka, M. (1996). Focal adhesions, contractility, and signaling. *Annu. Rev. Cell Dev. Biol.* 12, 463-519. 10.1146/annurev.cellbio.12.1.463 8970735

[JCS263580C8] Burridge, K. and Connell, L. (1983). A new protein of adhesion plaques and ruffling membranes. *J. Cell Biol.* 97, 359-367. 10.1083/jcb.97.2.359 6684120 PMC2112532

[JCS263580C9] Byron, A., Humphries, J. D., Askari, J. A., Craig, S. E., Mould, A. P. and Humphries, M. J. (2009). Anti-integrin monoclonal antibodies. *J. Cell Sci.* 122, 4009-4011. 10.1242/jcs.056770 19910492 PMC3329622

[JCS263580C10] Capaldo, C. T., Farkas, A. E., Hilgarth, R. S., Krug, S. M., Wolf, M. F., Benedik, J. K., Fromm, M., Koval, M., Parkos, C. and Nusrat, A. (2014). Proinflammatory cytokine-induced tight junction remodeling through dynamic self-assembly of claudins. *Mol. Biol. Cell* 25, 2710-2719. 10.1091/mbc.E14-02-0773 25031428 PMC4161507

[JCS263580C11] Ciobanasu, C., Faivre, B. and Le Clainche, C. (2013). Integrating actin dynamics, mechanotransduction and integrin activation: the multiple functions of actin binding proteins in focal adhesions. *Eur. J. Cell Biol.* 92, 339-348. 10.1016/j.ejcb.2013.10.009 24252517

[JCS263580C12] Citi, S., Fromm, M., Furuse, M., Gonzalez-Mariscal, L., Nusrat, A., Tsukita, S. and Turner, J. R. (2024). A short guide to the tight junction. *J. Cell Sci.* 137, jcs261776. 10.1242/jcs.261776 38712627 PMC11128289

[JCS263580C13] Cruz, N., Alvarez, X., Specian, R. D., Berg, R. D. and Deitch, E. A. (1994). Role of mucin, mannose, and beta-1 integrin receptors in Escherichia coli translocation across Caco-2 cell monolayers. *Shock* 2, 121-126. 10.1097/00024382-199408000-00007 7537165

[JCS263580C14] Dash, T. K. and Konkimalla, V. B. (2012). Poly-small je, Ukrainian-caprolactone based formulations for drug delivery and tissue engineering: a review. *J. Control. Release* 158, 15-33. 10.1016/j.jconrel.2011.09.064 21963774

[JCS263580C15] Den Beste, K. A., Hoddeson, E. K., Parkos, C. A., Nusrat, A. and Wise, S. K. (2013). Epithelial permeability alterations in an in vitro air-liquid interface model of allergic fungal rhinosinusitis. *Int. Forum Allergy Rhinol.* 3, 19-25. 10.1002/alr.21077 22927233 PMC3511593

[JCS263580C16] Ding, L., Wang, L., Sui, L., Zhao, H., Xu, X., Li, T., Wang, X., Li, W., Zhou, P. and Kong, L. (2016). Claudin-7 indirectly regulates the integrin/FAK signaling pathway in human colon cancer tissue. *J. Hum. Genet.* 61, 711-720. 10.1038/jhg.2016.35 27121327

[JCS263580C17] Finbloom, J. A., Demaree, B., Abate, A. R. and Desai, T. A. (2020). Networks of high aspect ratio particles to direct colloidal assembly dynamics and cellular interactions. *Adv. Funct. Mater.* 30, 2005938. 10.1002/adfm.202005938 33250685 PMC7687842

[JCS263580C18] Fredriksson, K., Van Itallie, C. M., Aponte, A., Gucek, M., Tietgens, A. J. and Anderson, J. M. (2015). Proteomic analysis of proteins surrounding occludin and claudin-4 reveals their proximity to signaling and trafficking networks. *PLoS ONE* 10, e0117074. 10.1371/journal.pone.0117074 25789658 PMC4366163

[JCS263580C19] Garbi, C., Negri, R., Cali, G. and Nitsch, L. (1996). Collagen interaction with apically expressed beta 1 integrins: loss of transepithelial resistance and reorganization of cultured thyroid cell monolayer. *Eur. J. Cell Biol.* 69, 64-75. 8825025

[JCS263580C20] Gonschior, H., Schmied, C., Van Der Veen, R. E., Eichhorst, J., Himmerkus, N., Piontek, J., Gunzel, D., Bleich, M., Furuse, M., Haucke, V. et al. (2022). Nanoscale segregation of channel and barrier claudins enables paracellular ion flux. *Nat. Commun.* 13, 4985. 10.1038/s41467-022-32533-4 36008380 PMC9411157

[JCS263580C21] Gonzalez-Mariscal, L., Avila-Flores, A. and Betanzos, A. (2001). The relationship between structure and function of tight junctions. In: *Tight Junctions*, 2nd edn. (ed. J. M. Anderson and M. Cereijido), pp. 89-120. Boca Raton, FL: CRC Press.

[JCS263580C22] Hall, D. E., Reichardt, L. F., Crowley, E., Holley, B., Moezzi, H., Sonnenberg, A. and Damsky, C. H. (1990). The alpha 1/beta 1 and alpha 6/beta 1 integrin heterodimers mediate cell attachment to distinct sites on laminin. *J. Cell Biol.* 110, 2175-2184. 10.1083/jcb.110.6.2175 2351695 PMC2116113

[JCS263580C23] He, W. Q., Wang, J., Sheng, J. Y., Zha, J. M., Graham, W. V. and Turner, J. R. (2020). Contributions of myosin light chain kinase to regulation of epithelial paracellular permeability and mucosal homeostasis. *Int. J. Mol. Sci.* 21, 993. 10.3390/ijms21030993 32028590 PMC7037368

[JCS263580C24] Higashi, T. and Chiba, H. (2020). Molecular organization, regulation and function of tricellular junctions. *Biochim. Biophys. Acta Biomembr.* 1862, 183143. 10.1016/j.bbamem.2019.183143 31812626

[JCS263580C25] Horton, E. R., Astudillo, P., Humphries, M. J. and Humphries, J. D. (2016). Mechanosensitivity of integrin adhesion complexes: role of the consensus adhesome. *Exp. Cell Res.* 343, 7-13. 10.1016/j.yexcr.2015.10.025 26515553

[JCS263580C26] Horwitz, A., Duggan, K., Buck, C., Beckerle, M. C. and Burridge, K. (1986). Interaction of plasma membrane fibronectin receptor with talin--a transmembrane linkage. *Nature* 320, 531-533. 10.1038/320531a0 2938015

[JCS263580C27] Huang, X., Shi, X., Hansen, M. E., Setiady, I., Nemeth, C. L., Celli, A., Huang, B., Mauro, T., Koval, M. and Desai, T. A. (2020). Nanotopography enhances dynamic remodeling of tight junction proteins through cytosolic liquid complexes. *ACS Nano* 14, 13192-13202. 10.1021/acsnano.0c04866 32940450 PMC7606830

[JCS263580C28] Humphries, J. D., Chastney, M. R., Askari, J. A. and Humphries, M. J. (2019). Signal transduction via integrin adhesion complexes. *Curr. Opin. Cell Biol.* 56, 14-21. 10.1016/j.ceb.2018.08.004 30195153

[JCS263580C29] Hynes, R. O. (2002). Integrins: bidirectional, allosteric signaling machines. *Cell* 110, 673-687. 10.1016/S0092-8674(02)00971-6 12297042

[JCS263580C30] Ivanov, A. I., Parkos, C. A. and Nusrat, A. (2010). Cytoskeletal regulation of epithelial barrier function during inflammation. *Am. J. Pathol.* 177, 512-524. 10.2353/ajpath.2010.100168 20581053 PMC2913378

[JCS263580C31] Jin, Y. and Blikslager, A. T. (2016). Myosin light chain kinase mediates intestinal barrier dysfunction via occludin endocytosis during anoxia/reoxygenation injury. *Am. J. Physiol. Cell Physiol.* 311, C996-C1004. 10.1152/ajpcell.00113.2016 27760753

[JCS263580C32] Jou, T. S., Schneeberger, E. E. and Nelson, W. J. (1998). Structural and functional regulation of tight junctions by RhoA and Rac1 small GTPases. *J. Cell Biol.* 142, 101-115. 10.1083/jcb.142.1.101 9660866 PMC2133025

[JCS263580C33] Kadry, Y. A. and Calderwood, D. A. (2020). Chapter 22: structural and signaling functions of integrins. *Biochim. Biophys. Acta Biomembr.* 1862, 183206. 10.1016/j.bbamem.2020.183206 31991120 PMC7063833

[JCS263580C34] Kam, K. R., Walsh, L. A., Bock, S. M., Koval, M., Fischer, K. E., Ross, R. F. and Desai, T. A. (2013). Nanostructure-mediated transport of biologics across epithelial tissue: enhancing permeability via nanotopography. *Nano Lett.* 13, 164-171. 10.1021/nl3037799 23186530 PMC4418930

[JCS263580C35] Kim, D. H., Lu, Q. and Chen, Y. H. (2019). Claudin-7 modulates cell-matrix adhesion that controls cell migration, invasion and attachment of human HCC827 lung cancer cells. *Oncol. Lett.* 17, 2890-2896. 10.3892/ol.2019.9909 30854065 PMC6365970

[JCS263580C36] Krause, G., Winkler, L., Mueller, S. L., Haseloff, R. F., Piontek, J. and Blasig, I. E. (2008). Structure and function of claudins. *Biochim. Biophys. Acta* 1778, 631-645. 10.1016/j.bbamem.2007.10.018 18036336

[JCS263580C37] Kummer, D., Steinbacher, T., Tholmann, S., Schwietzer, M. F., Hartmann, C., Horenkamp, S., Demuth, S., Peddibhotla, S. S. D., Brinkmann, F., Kemper, B. et al. (2022). A JAM-A-tetraspanin-alphavbeta5 integrin complex regulates contact inhibition of locomotion. *J. Cell Biol.* 221, e202105147. 10.1083/jcb.202105147 35293964 PMC8931538

[JCS263580C38] Lamson, N. G., Berger, A., Fein, K. C. and Whitehead, K. A. (2020). Anionic nanoparticles enable the oral delivery of proteins by enhancing intestinal permeability. *Nat. Biomed. Eng.* 4, 84-96. 10.1038/s41551-019-0465-5 31686002 PMC7461704

[JCS263580C39] Li, J. and Springer, T. A. (2017). Integrin extension enables ultrasensitive regulation by cytoskeletal force. *Proc. Natl. Acad. Sci. USA* 114, 4685-4690. 10.1073/pnas.1704171114 28416675 PMC5422820

[JCS263580C40] Lu, Z., Kim, D. H., Fan, J., Lu, Q., Verbanac, K., Ding, L., Renegar, R. and Chen, Y. H. (2015). A non-tight junction function of claudin-7-Interaction with integrin signaling in suppressing lung cancer cell proliferation and detachment. *Mol. Cancer* 14, 120. 10.1186/s12943-015-0387-0 26081244 PMC4470020

[JCS263580C41] Luo, B. H., Carman, C. V. and Springer, T. A. (2007). Structural basis of integrin regulation and signaling. *Annu. Rev. Immunol.* 25, 619-647. 10.1146/annurev.immunol.25.022106.141618 17201681 PMC1952532

[JCS263580C42] Luque, A., Gomez, M., Puzon, W., Takada, Y., Sanchez-Madrid, F. and Cabanas, C. (1996). Activated conformations of very late activation integrins detected by a group of antibodies (HUTS) specific for a novel regulatory region (355-425) of the common beta 1 chain. *J. Biol. Chem.* 271, 11067-11075. 10.1074/jbc.271.19.11067 8626649

[JCS263580C43] Lynn, K. S., Peterson, R. J. and Koval, M. (2020). Ruffles and spikes: control of tight junction morphology and permeability by claudins. *Biochim. Biophys. Acta Biomembr.* 1862, 183339. 10.1016/j.bbamem.2020.183339 32389670 PMC7299829

[JCS263580C44] Mould, A. P., Garratt, A. N., Askari, J. A., Akiyama, S. K. and Humphries, M. J. (1995). Identification of a novel anti-integrin monoclonal antibody that recognises a ligand-induced binding site epitope on the beta 1 subunit. *FEBS Lett.* 363, 118-122. 10.1016/0014-5793(95)00301-o 7537221

[JCS263580C45] Mould, A. P., Akiyama, S. K. and Humphries, M. J. (1996). The inhibitory anti-beta1 integrin monoclonal antibody 13 recognizes an epitope that is attenuated by ligand occupancy. Evidence for allosteric inhibition of integrin function. *J. Biol. Chem.* 271, 20365-20374. 10.1074/jbc.271.34.20365 8702772

[JCS263580C46] Park, C. C., Zhang, H. J., Yao, E. S., Park, C. J. and Bissell, M. J. (2008). Beta1 integrin inhibition dramatically enhances radiotherapy efficacy in human breast cancer xenografts. *Cancer Res.* 68, 4398-4405. 10.1158/0008-5472.CAN-07-6390 18519702 PMC3719863

[JCS263580C47] Peterson, R. J. and Koval, M. (2021). Above the matrix: functional roles for apically localized integrins. *Front. Cell Dev. Biol.* 9, 699407. 10.3389/fcell.2021.699407 34485286 PMC8414885

[JCS263580C48] Pulous, F. E., Grimsley-Myers, C. M., Kansal, S., Kowalczyk, A. P. and Petrich, B. G. (2019). Talin-dependent integrin activation regulates VE-cadherin localization and endothelial cell barrier function. *Circ. Res.* 124, 891-903. 10.1161/CIRCRESAHA.118.314560 30707047 PMC6521868

[JCS263580C49] Quiros, M. and Nusrat, A. (2014). RhoGTPases, actomyosin signaling and regulation of the epithelial Apical Junctional Complex. *Semin. Cell Dev. Biol.* 36, 194-203. 10.1016/j.semcdb.2014.09.003 25223584 PMC5054512

[JCS263580C50] Rouaud, F., Sluysmans, S., Flinois, A., Shah, J., Vasileva, E. and Citi, S. (2020). Scaffolding proteins of vertebrate apical junctions: structure, functions and biophysics. *Biochim. Biophys. Acta Biomembr.* 1862, 183399. 10.1016/j.bbamem.2020.183399 32553946

[JCS263580C51] Saeedi, B. J., Kao, D. J., Kitzenberg, D. A., Dobrinskikh, E., Schwisow, K. D., Masterson, J. C., Kendrick, A. A., Kelly, C. J., Bayless, A. J., Kominsky, D. J. et al. (2015). HIF-dependent regulation of claudin-1 is central to intestinal epithelial tight junction integrity. *Mol. Biol. Cell* 26, 2252-2262. 10.1091/mbc.E14-07-1194 25904334 PMC4462943

[JCS263580C52] Samak, G., Aggarwal, S. and Rao, R. K. (2011). ERK is involved in EGF-mediated protection of tight junctions, but not adherens junctions, in acetaldehyde-treated Caco-2 cell monolayers. *Am. J. Physiol. Gastrointest. Liver Physiol.* 301, G50-G59. 10.1152/ajpgi.00494.2010 21474650 PMC3129938

[JCS263580C53] Samak, G., Gangwar, R., Crosby, L. M., Desai, L. P., Wilhelm, K., Waters, C. M. and Rao, R. (2014). Cyclic stretch disrupts apical junctional complexes in Caco-2 cell monolayers by a JNK-2-, c-Src-, and MLCK-dependent mechanism. *Am. J. Physiol. Gastrointest. Liver Physiol.* 306, G947-G958. 10.1152/ajpgi.00396.2013 24722904 PMC4042113

[JCS263580C54] Sanders, M. A. and Basson, M. D. (2004). Collagen IV regulates Caco-2 migration and ERK activation via alpha1beta1- and alpha2beta1-integrin-dependent Src kinase activation. *Am. J. Physiol. Gastrointest. Liver Physiol.* 286, G547-G557. 10.1152/ajpgi.00262.2003 14604860

[JCS263580C55] Schneider, C. A., Rasband, W. S. and Eliceiri, K. W. (2012). NIH Image to ImageJ: 25 years of image analysis. *Nat. Methods* 9, 671-675. 10.1038/nmeth.2089 22930834 PMC5554542

[JCS263580C56] Schwartz, M. A. (2010). Integrins and extracellular matrix in mechanotransduction. *Cold Spring Harb. Perspect. Biol.* 2, a005066. 10.1101/cshperspect.a005066 21084386 PMC2982167

[JCS263580C57] Shen, B. Q., Xu, K., Liu, L., Raab, H., Bhakta, S., Kenrick, M., Parsons-Reponte, K. L., Tien, J., Yu, S. F., Mai, E. et al. (2012). Conjugation site modulates the in vivo stability and therapeutic activity of antibody-drug conjugates. *Nat. Biotechnol.* 30, 184-189. 10.1038/nbt.2108 22267010

[JCS263580C58] Spiess, M., Hernandez-Varas, P., Oddone, A., Olofsson, H., Blom, H., Waithe, D., Lock, J. G., Lakadamyali, M. and Stromblad, S. (2018). Active and inactive beta1 integrins segregate into distinct nanoclusters in focal adhesions. *J. Cell Biol.* 217, 1929-1940. 10.1083/jcb.201707075 29632027 PMC5987715

[JCS263580C59] Starchenko, A., Graves-Deal, R., Yang, Y. P., Li, C., Zent, R., Singh, B. and Coffey, R. J. (2017). Clustering of integrin alpha5 at the lateral membrane restores epithelial polarity in invasive colorectal cancer cells. *Mol. Biol. Cell* 28, 1288-1300. 10.1091/mbc.E16-12-0852 28356422 PMC5426844

[JCS263580C60] Stewart, T., Koval, W. T., Molina, S. A., Bock, S. M., Lillard, J. W., Jr, Ross, R. F., Desai, T. A. and Koval, M. (2017). Calibrated flux measurements reveal a nanostructure-stimulated transcytotic pathway. *Exp. Cell Res.* 355, 153-161. 10.1016/j.yexcr.2017.03.065 28390677 PMC5501187

[JCS263580C61] Su, Y., Xia, W., Li, J., Walz, T., Humphries, M. J., Vestweber, D., Cabanas, C., Lu, C. and Springer, T. A. (2016). Relating conformation to function in integrin α5β1. *Proc. Natl. Acad. Sci. USA* 113, E3872-E3881. 10.1073/pnas.1605074113 27317747 PMC4941492

[JCS263580C62] Sun, Z., Costell, M. and Fassler, R. (2019). Integrin activation by talin, kindlin and mechanical forces. *Nat. Cell Biol.* 21, 25-31. 10.1038/s41556-018-0234-9 30602766

[JCS263580C63] Sun, D., Luvalle-Burke, I., Pombo-Garcia, K. and Honigmann, A. (2022). Biomolecular condensates in epithelial junctions. *Curr. Opin. Cell Biol.* 77, 102089. 10.1016/j.ceb.2022.102089 35696872

[JCS263580C64] Tafazoli, F., Holmstrom, A., Forsberg, A. and Magnusson, K. E. (2000). Apically exposed, tight junction-associated beta1-integrins allow binding and YopE-mediated perturbation of epithelial barriers by wild-type Yersinia bacteria. *Infect. Immun.* 68, 5335-5343. 10.1128/IAI.68.9.5335-5343.2000 10948163 PMC101797

[JCS263580C65] Takada, Y. and Puzon, W. (1993). Identification of a regulatory region of integrin beta 1 subunit using activating and inhibiting antibodies. *J. Biol. Chem.* 268, 17597-17601. 10.1016/S0021-9258(19)85374-7 7688727

[JCS263580C66] Takagi, J. and Springer, T. A. (2002). Integrin activation and structural rearrangement. *Immunol. Rev.* 186, 141-163. 10.1034/j.1600-065x.2002.18613.x 12234369

[JCS263580C67] Takagi, J., Petre, B. M., Walz, T. and Springer, T. A. (2002). Global conformational rearrangements in integrin extracellular domains in outside-in and inside-out signaling. *Cell* 110, 599-511. 10.1016/s0092-8674(02)00935-2 12230977

[JCS263580C68] Tholmann, S., Seebach, J., Otani, T., Florin, L., Schnittler, H., Gerke, V., Furuse, M. and Ebnet, K. (2022). JAM-A interacts with alpha3beta1 integrin and tetraspanins CD151 and CD9 to regulate collective cell migration of polarized epithelial cells. *Cell. Mol. Life Sci.* 79, 88. 10.1007/s00018-022-04140-5 35067832 PMC8784505

[JCS263580C69] Tokuda, S., Higashi, T. and Furuse, M. (2014). ZO-1 knockout by TALEN-mediated gene targeting in MDCK cells: involvement of ZO-1 in the regulation of cytoskeleton and cell shape. *PLoS ONE* 9, e104994. 10.1371/journal.pone.0104994 25157572 PMC4144852

[JCS263580C70] Tornavaca, O., Chia, M., Dufton, N., Almagro, L. O., Conway, D. E., Randi, A. M., Schwartz, M. A., Matter, K. and Balda, M. S. (2015). ZO-1 controls endothelial adherens junctions, cell-cell tension, angiogenesis, and barrier formation. *J. Cell Biol.* 208, 821-838. 10.1083/jcb.201404140 25753039 PMC4362456

[JCS263580C71] Trevino, T. N. and Lutz, S. E. (2022). Matrix proteins plug a hole: how pericytes suppress blood brain barrier transcytosis. *Neuron* 110, 1601-1603. 10.1016/j.neuron.2022.04.011 35588710 PMC10015614

[JCS263580C72] Tsuchida, J., Ueki, S., Saito, Y. and Takagi, J. (1997). Classification of ‘activation’ antibodies against integrin beta1 chain. *FEBS Lett.* 416, 212-216. 10.1016/s0014-5793(97)01206-4 9369217

[JCS263580C73] Turner, J. R., Rill, B. K., Carlson, S. L., Carnes, D., Kerner, R., Mrsny, R. J. and Madara, J. L. (1997). Physiological regulation of epithelial tight junctions is associated with myosin light-chain phosphorylation. *Am. J. Physiol.* 273, C1378-C1385. 10.1152/ajpcell.1997.273.4.C1378 9357784

[JCS263580C74] Walsh, S. V., Hopkins, A. M., Chen, J., Narumiya, S., Parkos, C. A. and Nusrat, A. (2001). Rho kinase regulates tight junction function and is necessary for tight junction assembly in polarized intestinal epithelia. *Gastroenterology* 121, 566-579. 10.1053/gast.2001.27060 11522741

[JCS263580C75] Walsh, L., Ryu, J., Bock, S., Koval, M., Mauro, T., Ross, R. and Desai, T. (2015). Nanotopography facilitates in vivo transdermal delivery of high molecular weight therapeutics through an integrin-dependent mechanism. *Nano Lett.* 15, 2434-2441. 10.1021/nl504829f 25790174 PMC4478088

[JCS263580C76] Wang, Z., Symons, J. M., Goldstein, S. L., McDonald, A., Miner, J. H. and Kreidberg, J. A. (1999). (Alpha)3(beta)1 integrin regulates epithelial cytoskeletal organization. *J. Cell Sci.* 112, 2925-2935. 10.1242/jcs.112.17.2925 10444387

[JCS263580C77] Werb, Z., Tremble, P. M., Behrendtsen, O., Crowley, E. and Damsky, C. H. (1989). Signal transduction through the fibronectin receptor induces collagenase and stromelysin gene expression. *J. Cell Biol.* 109, 877-889. 10.1083/jcb.109.2.877 2547805 PMC2115739

[JCS263580C78] Yanez-Mo, M., Tejedor, R., Rousselle, P. and Sanchez-Madrid, F. (2001). Tetraspanins in intercellular adhesion of polarized epithelial cells: spatial and functional relationship to integrins and cadherins. *J. Cell Sci.* 114, 577-587. 10.1242/jcs.114.3.577 11171326

[JCS263580C79] Ye, F., Kim, C. and Ginsberg, M. H. (2012). Reconstruction of integrin activation. *Blood* 119, 26-33. 10.1182/blood-2011-04-292128 21921044 PMC3251231

[JCS263580C80] Zamecnik, C. R., Levy, E. S., Lowe, M. M., Zirak, B., Rosenblum, M. D. and Desai, T. A. (2020). An injectable cytokine trap for local treatment of autoimmune disease. *Biomaterials* 230, 119626. 10.1016/j.biomaterials.2019.119626 31753473 PMC6930339

[JCS263580C81] Zamecnik, C. R., Lowe, M. M., Patterson, D. M., Rosenblum, M. D. and Desai, T. A. (2017). Injectable polymeric cytokine-binding nanowires are effective tissue-specific immunomodulators. *ACS Nano* 11, 11433-11440. 10.1021/acsnano.7b06094 29124929 PMC5709211

