# Supplementary material for: Apical integrins as a switchable target to regulate the epithelial barrier
Source: J Cell Sci. 2024 Dec 20;137(24):jcs263580. doi: 10.1242/jcs.263580 (PMC11795292; doi:10.1242/jcs.263580)
Supplement: Supplementary information [file joces-137-263580-s1.pdf]

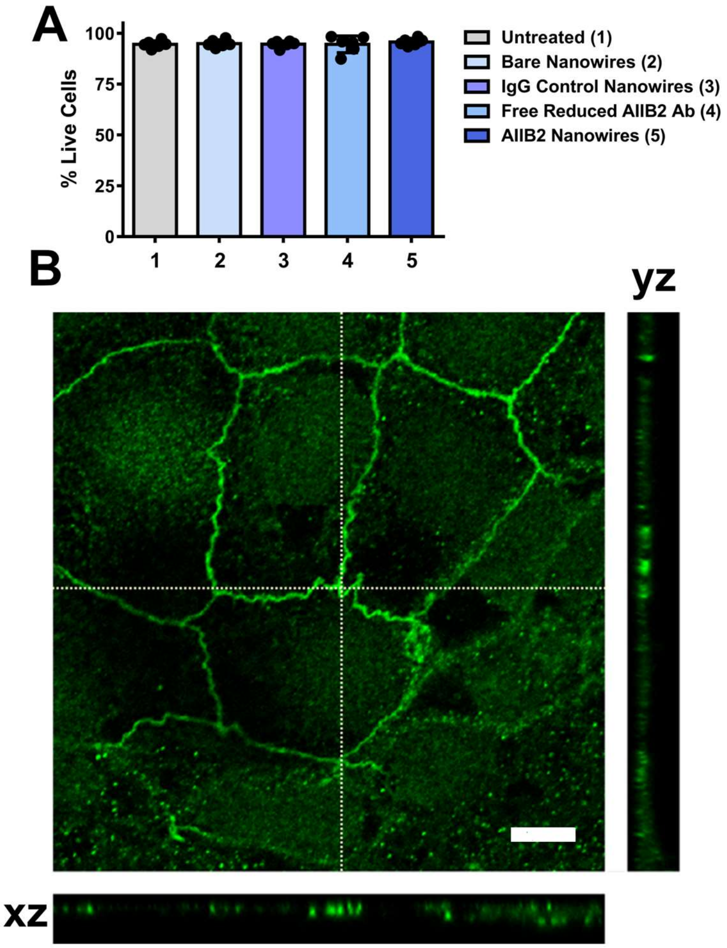

Fig. S1. Cell viability and tight junction orientation. (A) We used calcein as a measure to ensure that changes in barrier function were not a result of cell death. Graph depicts quantification of the percent of viable Caco-2 cells 2 h after treatment visualized by calcein-AM. Each data point is the number of viable cells in a field of view (n=2 biological replicates, 3 fields of view each). p>0.9999, significance was determined by one-way ANOVA with Bonferroni's correction for multiple comparisons. **(B)** Caco-2 cells were treated for 2 h with AIIB2-conjugated nanowires, fixed, stained for ZO-1 and then imaged using 3D confocal immunofluorescence microscopy. Location of the xz and yz sections are denoted by the dashed lines in the xy projection. Bar = 10 µm. Junction ruffling induced by AIIB2 nanowires did not alter the apical/lateral localization of ZO-1, which was in a plane comparable to non-ruffled junctions.

Journal of Cell Science • Supplementary information

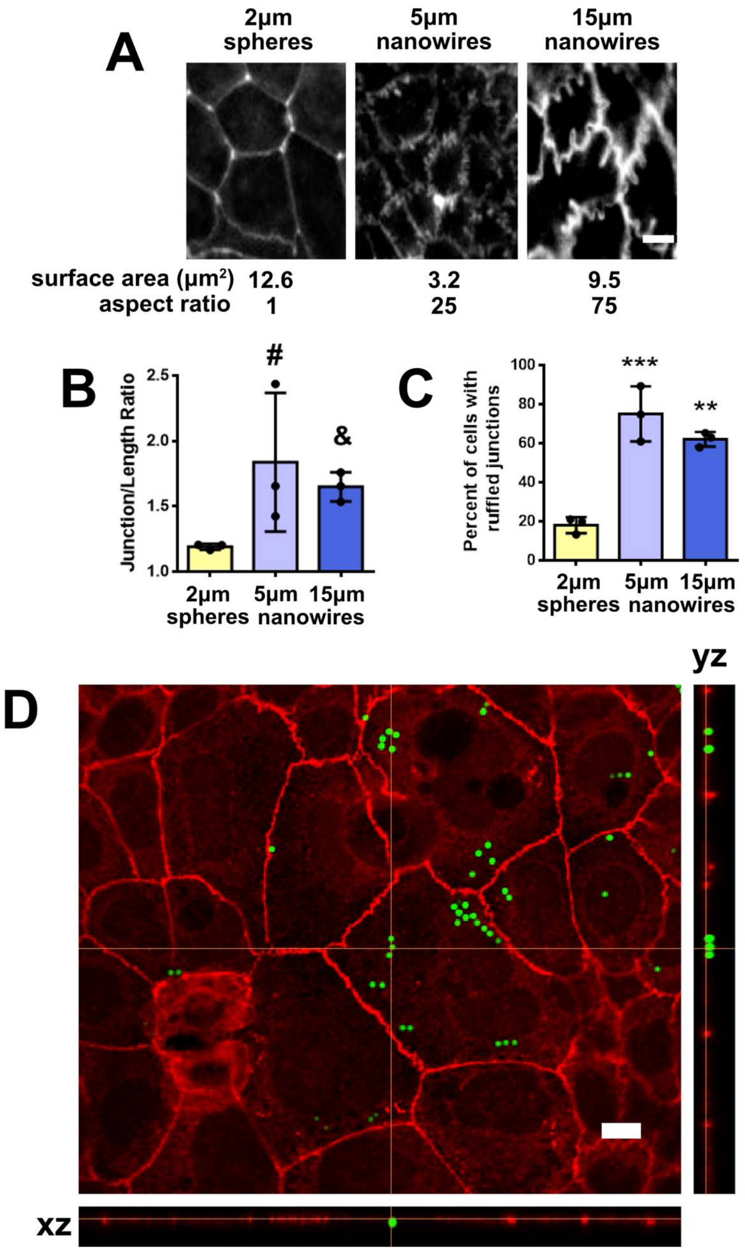

Fig. S2. ZO-1 ruffling depends on the aspect ratio of AIIB2 nanoparticles. (A) Representative immunofluorescence images of Caco-2 cells labeled with ZO-1 2 h after treatment with AIIB2 anti-integrin microspheres, 5 µm or 15 µm nanowires. Bar, 10 µm. **(B)** Quantification of junction/length ratios for each treatment, data displayed as mean ratio per field of view ± SEM (n = 25 measurements per field of view from 3 fields of view from 1 biological replicate. ****p<0.0001 **(C)** Quantification of percent cells in a field of view with one or more

Journal of Cell Science • Supplementary information

ruffled junctions displayed as percent ± SEM (n = 3 fields of view). **p=0.0026, ***p=0.0006. Significance determined by one-way ANOVA with Bonferroni's correction for multiple comparisons. **(D)** Caco-2 cells were treated for 2 h with AIIB2 microspheres, fixed, stained for ZO-1 and then imaged using 3D confocal immunofluorescence microscopy. Location of the xz and yz sections are denoted by the orange lines in the xy projection. Bar = 10 μm. AIIB2 microspheres were aligned with ZO-1 indicating that they were not internalized by the cells.

Journal of Cell Science • Supplementary information

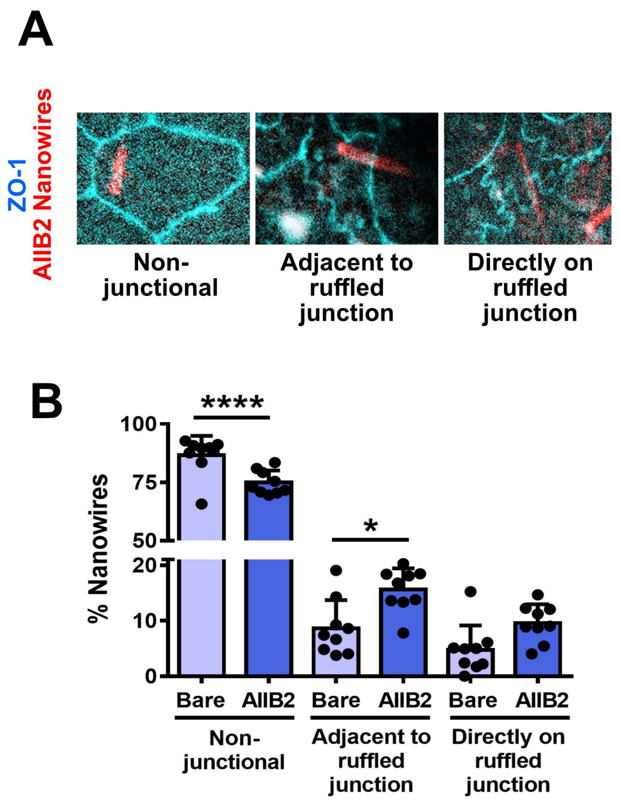

Fig. S3. Localization of nanowires relative to changes in tight junction morphology. (A) Nanowire (red) localization either represented as either non junctional, in direct contact with ruffles or in contact with junctions adjacent to ruffled junctions, using ZO-1 as a tight junction marker (cyan). **(B)** Localization was quantified, where each data point represents the amount of junctionally localized nanowires as a percentage of the total cell associated nanowires in each field of view. Data is displayed as mean + SD (n = 3 fields of view from 3 slides per condition), * p = 0.022, **** p < 0.0001. Significance was determined by one-way ANOVA with Bonferroni correction for multiple comparisons.

Journal of Cell Science • Supplementary information

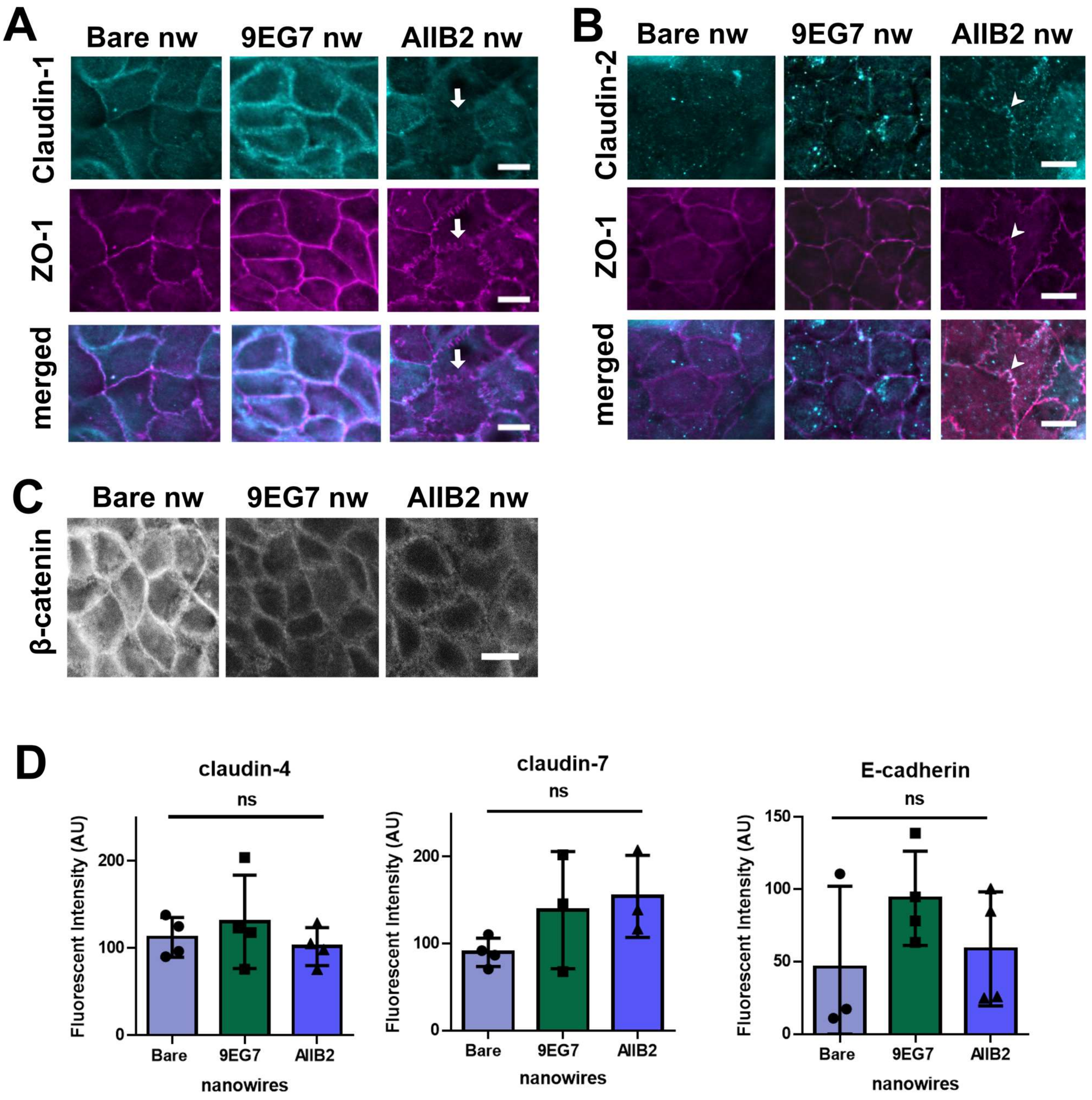

Fig. S4. Effect of targeting apical integrins with AIIB2 nanowires on junction morphology. In each case, Caco-2 cells were incubated with either bare, 9EG7 or AIIB2 nanowires for 2 h prior to fixation, staining and imaging. **(A)** Representative immunofluorescence images of Caco-2 cells co-labeled for claudin-1 (cyan) and ZO-1 (magenta). Arrows represent areas where claudin-1 does not colocalize with ruffled ZO-1. Bar, 10 µm. **(B)** Representative immunofluorescence images of Caco-2 cells co-labeled for claudin-2 (cyan) and ZO-1

Journal of Cell Science • Supplementary information

(magenta). Arrowheads show co-localization of claudin-2 with ZO-1. Bar, 10 µm. **(C)** Representative immunofluorescence images of Caco-2 cells labeled with β-catenin. Bar, 10 µm. **(D)** Immunofluorescence images were masked using an Otsu threshold to isolate junction associated signals and were then quantified for relative intensity using Image J. Shown are mean ± SD. Based on one-way-ANOVA, treatment with different classes of nanowires did not cause any significant differences in intensity.

Table S1. Primary antibodies used for immunofluorescence and fixation conditions.

Protein	Catalog number	Fixation method	Concentration
β-catenin	BD Biosciences, 610153	4% PFA only	1:500
Claudin-1	Thermo Fisher Scientific, 51-9000	4% PFA + methanol:acetone	1:500
Claudin-2	Thermo Fisher Scientific, 51-6100	4% PFA + methanol:acetone	1:100
Claudin-4	Thermo Fisher Scientific, 36-4800	4% PFA + methanol:acetone	1:100
Claudin-7	Thermo Fisher Scientific, 34-9100	4% PFA + methanol:acetone	1:500
E-cadherin	Cell Signaling Technology, 24E10	4% PFA + methanol:acetone	1:500
Pan-Talin	Sigma Aldrich, T3287 (clone 8d4)	4% PFA only	1:100
ZO-1	Thermo Fisher Scientific, 33-9100	4% PFA + methanol:acetone	1:1000

Journal of Cell Science • Supplementary information
